# A model study for the manufacture and validation of clinical-grade deciduous dental pulp stem cells for chronic liver fibrosis treatment

**DOI:** 10.1186/s13287-020-01630-w

**Published:** 2020-03-25

**Authors:** Tsuyoshi Iwanaka, Takayoshi Yamaza, Soichiro Sonoda, Koichiro Yoshimaru, Toshiharu Matsuura, Haruyoshi Yamaza, Shouichi Ohga, Yoshinao Oda, Tomoaki Taguchi

**Affiliations:** 1 Department of Pediatric Surgery, Kyushu University Graduate School of Medical Sciences, 3-1-1 Maidashi, Higashi-ku, Fukuoka, 812-8582 Japan; 2 Department of Molecular Cell Biology and Oral Anatomy, Kyushu University Graduate School of Dental Science, 3-1-1 Maidashi, Higashi-ku, Fukuoka, 812-8582 Japan; 3 Department of Pediatric Dentistry, Kyushu University Graduate School of Dental Science, 3-1-1 Maidashi, Higashi-ku, Fukuoka, 812-8582 Japan; 4 Department of Pediatrics, Kyushu University Graduate School of Medical Sciences, 3-1-1 Maidashi, Higashi-ku, Fukuoka, 812-8582 Japan; 5 Department of Anatomic Pathology, Kyushu University Graduate School of Medical Sciences, 3-1-1 Maidashi, Higashi-ku, Fukuoka, 812-8582 Japan

**Keywords:** Human deciduous pulp stem cells (hDPSCs), Clinical-grade, Manufacturing, Validation, Chronic liver fibrosis

## Abstract

**Background:**

Human deciduous pulp stem cells (hDPSCs) have remarkable stem cell potency associated with cell proliferation, mesenchymal multipotency, and immunosuppressive function and have shown beneficial effects in a variety of animal disease models. Recent studies demonstrated that hDPSCs exhibited in vivo anti-fibrotic and anti-inflammatory action and in vivo hepatogenic-associated liver regeneration, suggesting that hDPSCs may offer a promising source with great clinical demand for treating liver diseases. However, how to manufacture ex vivo large-scale clinical-grade hDPSCs with the appropriate quality, safety, and preclinical efficacy assurances remains unclear.

**Methods:**

We isolated hDPSCs from human deciduous dental pulp tissues formed by the colony-forming unit-fibroblast (CFU-F) method and expanded them under a xenogeneic-free and serum-free (XF/SF) condition; hDPSC products were subsequently stored by two-step banking including a master cell bank (MCB) and a working cell bank (WCB). The final products were directly thawed hDPSCs from the WCB. We tested the safety and quality check, stem cell properties, and preclinical potentials of final hDPSC products and hDPSC products in the MCB and WCB.

**Results:**

We optimized manufacturing procedures to isolate and expand hDPSC products under a XF/SF culture condition and established the MCB and the WCB. The final hDPSC products and hDPSC products in the MCB and WCB were validated the safety and quality including population doubling ability, chromosome stability, microorganism safety, and stem cell properties including morphology, cell surface marker expression, and multipotency. We also evaluated the in vivo immunogenicity and tumorigenicity and validated in vivo therapeutic efficacy for liver regeneration in a CCl_4_-induced chronic liver fibrosis mouse model in the final hDPSC products and hDPSC products in the WCB.

**Conclusion:**

The manufacture and quality control results indicated that the present procedure could produce sufficient numbers of clinical-grade hDPSC products from a tiny deciduous dental pulp tissue to enhance clinical application of hDPSC products in chronic liver fibrosis.

**Electronic supplementary material:**

The online version of this article (10.1186/s13287-020-01630-w) contains supplementary material, which is available to authorized users.

## Background

Human mesenchymal stem cells (MSCs) were first identified as non-hematopoietic lineage cells in the bone marrow and exhibited plastic adherent colony formation, including fibroblast-shaped cells, colony-forming unit-fibroblast (CFU-F) [1]. Because of the therapeutic benefit including self-replication, survival, multi-differentiation, and immunomodulation [2–4], MSC-based therapy has become a feasible and promising option for various refractory diseases [5]; MSCs have been tested in several clinical trials [6–8]. Large-scale ex vivo growth and a steady supply of clinical-grade MSCs is required for the wide range of clinical applications in human MSC-based tissue engineering and regenerative medicine. However, the production of clinical-grade human MSCs for patient treatment cannot be achieved using the simple laboratory-scale procedure, including the isolation and expansion of MSC for basic and translational studies. The ex vivo standard procedure for clinical-grade MSCs should be employed to produce clinical-grade MSC products with a defined quality and safety as well as an ensured steady supply [9, 10]. To produce final products with a defined quality and safety that are clinically utilized for patients, a validated standard operating procedure (SOP) needs to be established for the whole manufacturing and quality control process to produce clinical-grade MSCs [11]. Recently, MSC products have been commercially approved for treating patients with refractory graft-versus-host disease (GVHD) in Korea, Canada, New Zealand, and Japan [12, 13].

Diverse congenital and acquired liver diseases, such as liver cirrhosis, metabolic liver diseases, and hepatic carcinoma, eventually result in end-stage hepatic disorders including fulminant hepatitis and liver failure. Orthotopic liver transplantation (OLT) is the only radical option to recover from end-stage liver conditions [14]. However, there are several problems with OLT, such as long-term waiting and shortage of donor organs, surgical injuries, organ rejection, life-long use of immunosuppressive drugs, and high medical costs, which burden patients with end-stage liver disorders [15]. On the other hand, human primary hepatocyte transplantation may be a considerable alternative to OLT [16, 17], but the shortage of effective donor hepatocytes limits the clinical benefit of primary hepatocyte transplantation [18], indicating that a novel alternative therapeutic is required for patients with end-stage liver disorders. Recently, MSC-based therapy was considered as a potential option for refractory liver disease and was subsequently introduced into clinical trials [19, 20].

Human MSCs isolated from dental pulp tissue of deciduous teeth represent a feasible candidate for future MSC-based therapies. The human deciduous pulp stem cells (hDPSCs) exhibit remarkable stem cell potency, including cell proliferation, mesenchymal multipotency, and immunosuppressive function, in comparison with bone marrow-derived MSCs [21, 22]. Moreover, current xenogeneic transplant studies in a variety of animal disease models have provided beneficial evidence of the usefulness of hDPSCs [22, 23]. More recently, clinical studies reported the therapeutic use of hDPSCs for dental pulp regeneration in injured teeth [24]. Therefore, given recent studies of in vitro hepatogenic capacity [25] and the in vivo liver regenerative efficacy of hDPSCs [26], hDPSCs may offer a promising source with superior clinical efficacy and great clinical demand for treating liver diseases. However, it is unclear how to generate consistent, large-scale ex vivo clinical-grade hDPSCs using the current isolation and culture techniques. An incomplete understanding of the effectiveness of clinical-grade hDPSC products to treat liver disorders and the lack of a SOP to secure the quality and safety of clinical-grade hDPSCs remain to be addressed before introducing clinical-grade hDPSC products into clinical applications.

In this study, we established a practical and reliable procedure for the clinical-grade production of dental pulp stem cells from human deciduous teeth, hDPSCs, based on a CFU-F method. We optimized the manufacturing procedures to isolate, expand, and store clinical-grade hDPSC products under xenogeneic-free (XF) and serum-free (SF) (XF/SF) culture conditions. In addition, we evaluated the final clinical-grade hDPSC products, not only for in vitro quality and safety, but also for in vivo therapeutic efficacy for liver regeneration in a CCl_4_-induced chronic liver fibrosis mouse model to enhance clinical application of clinical-grade hDPSC products.

## Materials and methods

### Ethics statement and human subjects

Procedures for handling human samples were approved by the Kyushu University Institutional Review Board for Human Genome/Gene Research (Protocol Number: 393–01). We obtained written informed consent from each guardian on behalf of the child donors. All pediatric donors were clinically diagnosed as healthy without any symptoms and systemic diseases (Table 1). Human deciduous teeth, which were almost exfoliated, were extracted from the pediatric donors in the Department of Pediatric Dentistry of Kyushu University Hospital. All animal experiments in this study were approved by the Institutional Animal Care and Use Committee of Kyushu University (Protocol Number: A21-044-1). All methods were performed in accordance with the relevant guidelines and regulations.

**Table 1:** Summary of donor information

Donor	Sex	Age (years, months)	Tooth number	Medical history
#1	Male	6y3m	11	None
#2	Female	6y11m	21	None
#3	Male	7y0m	11	None
#4	Female	7y3m	11	None
#5	Male	7y7m	11	None
#6	Female	6y10m	21	None
#7	Female	8y0m	21	None
#8	Male	7y4m	21	None
#9	Male	6y10m	11	None
#10	Male	7y8m	11	None

Tooth numbers are determined by the Fédération Dentaire Internationale (FDI) tooth-numbering system

### Animals and cell lines

Wild-type C57BL/6J mice (male, 8 weeks old) were obtained from Charles River Laboratories Japan (Yokohama, Japan). The animals were maintained with free access to sterilized water and a standard chow MF diet (Oriental Yeast, Tokyo, Japan) under controlled environmental conditions with a 12-h light/12-h dark cycle. The human hepatoma-cell line HepG2 cells were provided from Riken BRC (Tsukuba, Japan) and were cultured according to the manufacturers’ instruction.

### Isolation of hDPSCs under XF conditions

All extracted deciduous teeth used in this study were quickly stored in 20 mL of Dulbecco’s modified Eagle’s medium (Thermo Fisher Scientific, Waltham, MA) alone at 4 °C and were transported from the clinic to laboratory bench at 4 °C within 24 h before we start to isolate hDPSCs. Isolation of hDPSCs was performed under XF conditions by a colony-forming unit-fibroblast (CFU-F) method [1]. The deciduous pulp tissue of deciduous teeth was removed and was minced with a surgical blade, followed by digestion with 3 mL of the XF tissue digestion regent Liberase™ MNP-S (0.45 Wünsch units/mL; Roche, Basel, Switzerland) diluted in sterilized Ca^2+^-free and Mg^2+^-free phosphate-buffered saline (PBS; Nacalai Tesque, Kyoto, Japan) at 37 °C for 30 min. Each digested suspension was passed through a 70-μm cell strainer (Corning, Corning, NY) into a 50-mL polystyrene tube (Corning) to obtain a single-cell suspension and was centrifuged at 770×*g* at 4 °C for 5 min in an Allegra® X-30R centrifuge machine (Beckman Coulter, Brea, CA) equipped with a SX4400 swinging rotor (Beckman Coulter). The single-cell suspension was seeded into a T-75 culture flask (Corning) with 10 mL of a MSC NutriStem® XF Medium (XFM; Biological Industries, Beit HaEmek, Israel) without antibiotics. Eighteen hours after the initial seeding, the culture flasks were washed twice with 1 mL of PBS (Nacalai Tesque) to remove floating cells and were further cultured for 10–14 days with 10 mL of XFM (Biological Industries). The cells were maintained at 37 °C with 5% CO_2_ in a Forma™ CO_2_ incubator (Thermo Fisher Scientific). Adherent colony formation was inspected daily and was confirmed using a Primovert inverted microscope (Carl Zeiss Microscopy, Jena, Germany).

### Cell passage and expansion of hDPSC products

Cultured medium was removed, and the culture flasks were washed twice with 1 mL of PBS (Nacalai Tesque). The hDPSC products were removed from the flask using 1 mL of a cell removal reagent TrypLE™ select without phenol red (Thermo Fisher Scientific) at 37 °C for 5 min. The removed hDPSC products were collected by centrifugation, as described above, and the cell pellet was diluted in XFM (1 mL; Biological Industries). The hDPSC products (0.25 × 10^6^ per flask) were seeded in a new T-75 flask (Corning) with XFM (10 mL; Biological Industries). When they were 70% confluent, the hDPSC products were passaged again to expand as described above. The medium was exchanged twice a week. The cells were maintained at 37 °C with 5% CO_2_ in a Forma™ CO_2_ incubator (Thermo Fisher Scientific) and were inspected daily using a Primovert inverted microscope (Carl Zeiss Microscopy).

### Cell counting of hDPSC products

A dye exclusion test with trypan blue (Bio-Rad Laboratories, Hercules, CA) was performed to determine the cell number. The number of the hDPSC products was counted with a TC20 cell counter (Bio-Rad Laboratories) using its counting slides (Bio-Rad Laboratories), according to the manufacturer’s instructions. The hDPSC products were counted in triplicate and the mean of the three measurements was adopted as the cell number.

### Cell freezing and thawing of hDPSC products

The hDPSC products were washed with 1 mL of PBS (Nacalai Tesque) twice and were treated with 1 mL of TrypLE™ select without phenol red (Thermo Fisher Scientific) at 37 °C for 5 min. The removed hDPSC products were mixed at a concentration of 1.0 × 10^6^/mL with a STEM CELL BANKER® cryomedium (Zenoak, Fukushima, Japan), followed by storage in cryogenic vials (Sumitomo Bakelite, Tokyo, Japan). The vials were kept for 4 h at − 80 °C in a CoolCell® LX ell freezing container (BioCision, Larkspur, CA) and then were packed in a cryogenic Nalgene® CryoBox™ System 100™ container (Thermo Fisher Scientific). Finally, the containers were stored in a LS6000 cryogenic refrigerator (Worthington, Columbus, OH) filled with liquid nitrogen.

The frozen hDPSC products were thawed with ThawSTAR® CFT2 (Astero, Bothell, WA) and were transferred to a 15-mL polystyrene tube (Corning) containing 4 mL of XFM (Biological Industries). The cell pellet was obtained by centrifugation using the same conditions as described above. The supernatant was removed and the cell pellet was suspended in 1 mL of XFM (Biological Industries). The number of hDPSC products in the suspension was counted as described above, and the hDPSC products (0.25 × 10^6^ per flask) were seeded in a T-75 flask (Corning). The medium was exchanged twice a week.

### Adherent colony formation assay of hDPSCs

Isolated cells (10 × 10^3^) from the deciduous dental pulp tissue were seeded into a 100-mm culture dish (Corning) and were cultured in 10 mL of XFM (Biological Industries) for 14 days. The culture dish was treated with 3 mL of 4% paraformaldehyde (Merck, Darmstadt, Germany) and 0.1% toluidine blue (Merck) in PBS (pH 7.4; Nacalai Tesque) for 18 h. After washing five times with 1 mL of PBS, the dish was air-dried and was imaged with a GT-X980 scanner (Epson, Suwa, Nagano). Numbers of CFU-F, which contained > 50 cells, were counted using a Primovert inverted microscope (Carl Zeiss Microscopy).

### Population doubling (PD) assay of hDPSC products

The P1 hDPSC products were sequentially passaged every 7 days under XF/SF conditions until they lost their dividing ability. The culture medium was changed every 3 days. The population doubling level (PDL) at each passage was calculated according to the equation: PDL = 3.32 (log [number of harvested cells at end of a passage] − log [number of seeded cells at initial of a passage]).

### Chromosomal analysis of hDPSC products

Chromosomal analysis of the hDPSC products, including chromosome numbers and G-banded karyotypes, was performed with Giemsa staining by LSI Medience Corporation (Tokyo, Japan).

### Microorganism tests of hDPSC products

Microorganism tests for endotoxin, Treponema, Mycoplasma, cytomegalovirus (CMV), hepatitis B virus (HBV), HCV, human immunodeficiency virus 1 (HIV-1), human T-cell leukemia virus type 1 (HTLV-1), and parvovirus were analyzed using the conditioned medium (CM) of the hDPSC products by LSI Medience Corporation (Tokyo, Japan).

### Characterization of hDPSC products

Characterization of the hDPSC products was examined according to recent studies [21, 22, 27], as described in the Supplementary Methods. As for cell morphology assay, hDPSC products in a T-75 flask (Corning) were imaged under a Primovert inverted microscope (Carl Zeiss Microscopy). The expression of MSC surface markers including human CD146, CD105, CD73, CD90, CD34, CD45, CD14, and human leukocyte antigen DR (HLA-DR), which were referred from a previous report [28], in hDPSC products was examined by flow cytometric analysis (FCM). The lineage-specific-gene expression was analyzed in the hDPSC products cultured under the specific inductive conditions into osteoblasts, chondrocytes, and adipocytes by quantitative reverse transcription polymerase chain reaction (qRT-PCR) assay. For secretome assay, hepatocyte growth factor (HGF), interleukin 6 (IL6), monocyte chemotactic protein 1 (MCP1), and sialic acid-binding immunoglobulin-type lectin 9 (SIGLEC9) in the CM of the hDPSC products were analyzed by enzyme-linked immunosorbent assay (ELISA).

### Hepatogenic differentiation of hDPSC products

Hepatogenic differentiation of the hDPSC products was induced according to a previously reported protocol [25] and analyzed by qRT-PCR assay, as described in the Supplementary Methods.

### *Stanniocalcin 1* (*STC1*) expression in hDPSC products

The hDPSC products were cultured according to a previously reported protocol [25]. Expression of *STC1* was analyzed in hDPSCs by qRT-PCR assay, as described in the Supplementary Methods.

### In vitro tumorigenicity of hDPSCs

The in vitro colony-forming ability of the hDPSC products was tested using a Cell Transformation Detection Assay (Merck) according to the manufacturer’s instructions. The hDPSC products (500 cells per well) were cultured in agar for 2 weeks, and the colony formation was analyzed under a Primovert microscope (Carl Zeiss). Telomerase activity in hDPSC products was tested using a telomerase repeat amplification protocol with a Telo *TAGGG* Telomerase PCR ELISA^PLUS^ (Merck) according to the manufacturer’s instructions, as described in the Supplementary Methods. Expression of *MYC* was analyzed in hDPSCs using qRT-PCR, as described in the Supplementary Methods. HepG2 cells were used as a positive control in all tests.

### Immunogenicity of hDPSCs

Immunogenic markers including HLAs (HLA-ABC, HLA-E, HLA-DR, and HAL-DQ), T-cell cofactors (CD80, CD86, and CD40), and a T-cell marker (CD3) in hDPSCs were analyzed by FCM. For T cell activity assay, a mixed culture experiment of the hDPSC products and human peripheral blood mononuclear cells (PBMNCs) was tested as in a previous report [22], as described in the Supplementary Methods.

### Transplantation of hDPSC products into chronically CCl_4_-injured mice

Wild-type mice at 9 weeks of age intraperitoneally received a CCl_4_ solution twice a week for 4 weeks. The CCl_4_ solution was freshly prepared CCl_4_ (0.5 mg/kg in olive oil [Wako Pure Chemicals]; CCl_4_:olive oil = 1:4 volume/volume). The 4-week-CCl_4_-treated mice received each donor-derived hDPSC products (0.1 × 10^6^/10 g body weight in PBS) via the spleen [26]. The hDPSC-transplanted mice were subsequently treated with CCl_4_. The age-matched mice that received olive oil and sham-operated CCl_4_ were used as negative and positive controls.

### Histological and immunohistochemical analyses of mouse liver tissues

The paraffin sections of mouse livers were treated with Picrosirius Red staining [26]. Immunohistochemical analysis was also tested to determine the localization of HLA-ABC, human hepatocyte antigen paraffin 1 (HepPar1), human albumin, and mouse actin, alpha 2, smooth muscle (ACTA2), as described in the Supplementary Methods. Immunohistochemical controls were tested using non-immune antibodies. All sections were observed under a BZ X-700 microscope (KEYENCE, Tokyo, Japan).

Five immunohistochemical sections were randomly selected and were analyzed for the percentage of fibrous tissue area or primary antibody-positive area using ImageJ software (NIH, Bethesda, MD, USA). Picrosirius Red-stained sections were also analyzed to score the amount of liver damage using Ishak scoring [29].

### Liver functional assays of mouse serum

Alanine aminotransferase (ALT), aspartate transferase (AST), and total bilirubin in mouse serum were measured by biochemical assays. Human albumin in mouse serum was analyzed by ELISA. Biochemical assays and ELISAs were performed as described in the Supplementary Methods.

### Liver fibrosis assay of mouse liver tissues

Gene expression of mouse collagen type I alpha 1 a (*Col1a1*) and *Acta2* was tested by qRT-PCR, as described in the Supplementary Methods.

### Statistical analysis

Statistical results are expressed as mean ± standard error of the mean (SEM) or mean ± standard deviation (SD). Multiple group comparisons were analyzed by one-way repeated-measures analysis of variance (ANOVA) followed by the Tukey post hoc test. Values of *P* < 0.05 were considered significant. All statistics were analyzed using JMP 11 (SAS Institute, Cary, NC).

## Results

### Establishment of a SOP for the manufacture and quality control of hDPSC production using CFU-F-derived multi-colonies under XF conditions

**Fig. 1:**
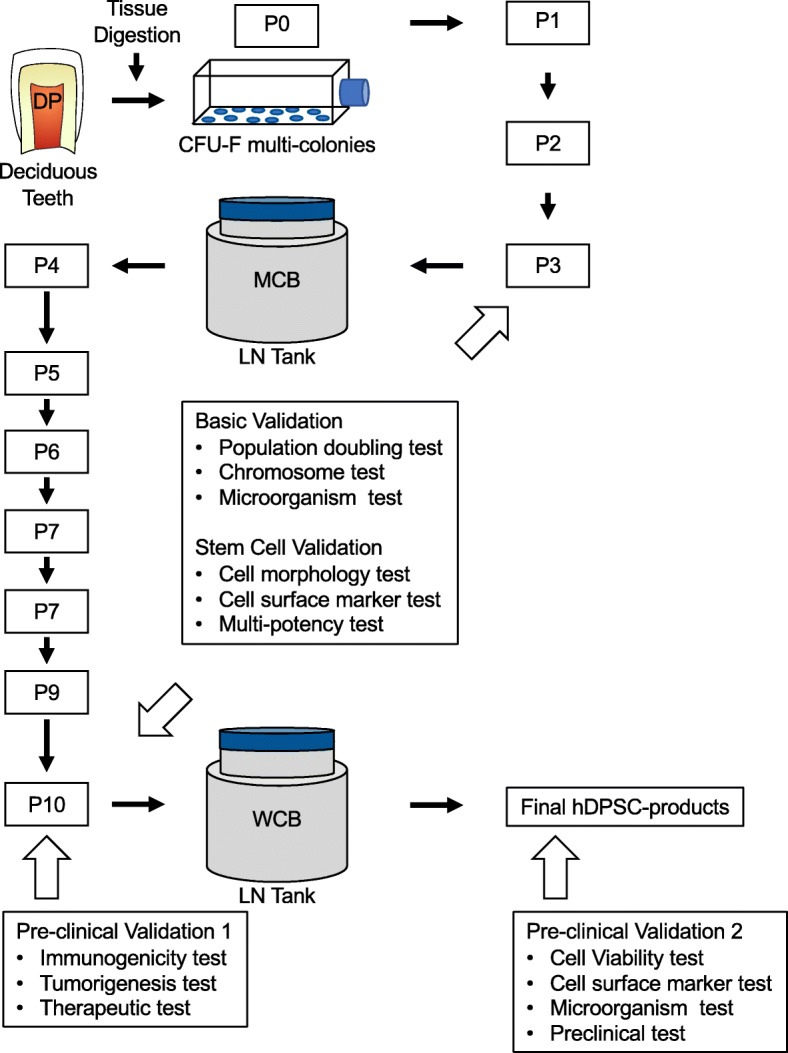
Schema of production and quality control checks of clinical-grade human deciduous pulp stem cells (hDPSCs). The present procedure for clinical-grade hDPSCs is composed of isolation of colony-forming unit-fibroblast (CFU-F)-based multi-colonies, culture steps, and two-step cell banking. Briefly, dental pulp tissues (DP) of deciduous teeth were extracted and were digested. The cell suspension was seeded into a T-75 culture flask, and the attached multi-colonies were obtained. The formed multi-colonies were collected and were passaged for further expansion. hDPSCs harvested at passages 3 (P3) and P10 were cryopreserved for an master cell bank (MCB) and a working cell bank (WCB), respectively, in a liquid nitrogen (LN) tank. Final hDPSC products were obtained from cells thawed from the WCB batch. To manufacture high numbers, safety, and quality of the final cell products for the patients, multi-step validation for quality, safety, and preclinical check was employed as indicated at each step. The final hDPSC products should meet all criteria. Full details are described in the “Results” and “Materials and methods” sections of this manuscript

The basic procedure of the present isolation and expansion of hDPSCs was similar to the original procedure [21, 22], but the difference was propagation to secure XF/SF conditions compared to the original procedure. We then optimized the laboratory-scale CFU-F protocol for large-scale expansion (Fig. 1). Remnant dental pulp tissues of deciduous teeth extracted from healthy donors were treated with a XF tissue digestion regent, and the single-cell suspension was seeded into a regular T-75 cm^2^ plastic culture flask with a commercially available XF medium. Eighteen hours after the initial seeding, any floating cells were removed from the flasks, and the attached cells on the bottom of the flasks were maintained to form adherent colonies in XF/SF conditions. The whole colony-forming cells were collected from a flask and were re-plated into a T-75 cm^2^ flask at a seeding density of 0.25 × 10^6^ per T-75 cm^2^ flask as P1. In the present SOP, for the cell expansion process, T-75 cm^2^ flasks were used and the seeding number was 0.25 × 10^6^ per T-75 cm^2^ flask at each passage. Cell passage was performed every 7 days. Cell storage steps for the master cell bank (MCB) and the working cell bank (WCB) were added to the present manufacturing process. Lastly, the final hDPSC products for patients were cataloged to provide a directory of the hDPSC products stored in WCB.

In the present SOP, several assurances for the quality, safety, and efficacy of hDPSC products were validated at each step, including the MCB, the WCB, and the final products. In the present procedure, the basic criteria included the total PDL 20, chromosomal stability, and microorganism safety and were measured for the WCB. To determine the timing for the WCB by the PDL 20 threshold, hDPSC products were prepared for a sequential passage. In the process of the sequential passage, the P3 hDPSC products were stored in the MCB to maintain the cells with the significantly high proliferation potential at an early stage for future manufacturing. If hDPSC products for the WCB storage failed to meet any of the basic criteria, they were discarded immediately. Only the hDPSC products that passed the basic criteria for the WCB, as well as the P3 hDPSC products for MCB, were employed to test further MSC criteria including cell morphology, surface marker analysis, and multipotency. The hDPSC products in the MCB and the WCB storages must meet all prerequisite criteria. If the cells failed to meet any of the criteria, they were discarded immediately. Moreover, the hDPSC products for the WCB storage were used for additional tests on the in vitro immunogenicity and tumorigenicity and were also tested in an in vitro hepatogenic capacity. The quality tests of the final hDPSC products were cell viability, MSC marker analysis, and microorganism tests. In vivo preclinical tests were also carried out to determine the therapeutic efficacy of the final hDPSC products, as well as hDPSC products in the WCB, in a chronic liver fibrosis mouse model.

### Primary validation of hDPSC products for MCB and WCB products

**Fig. 2:**
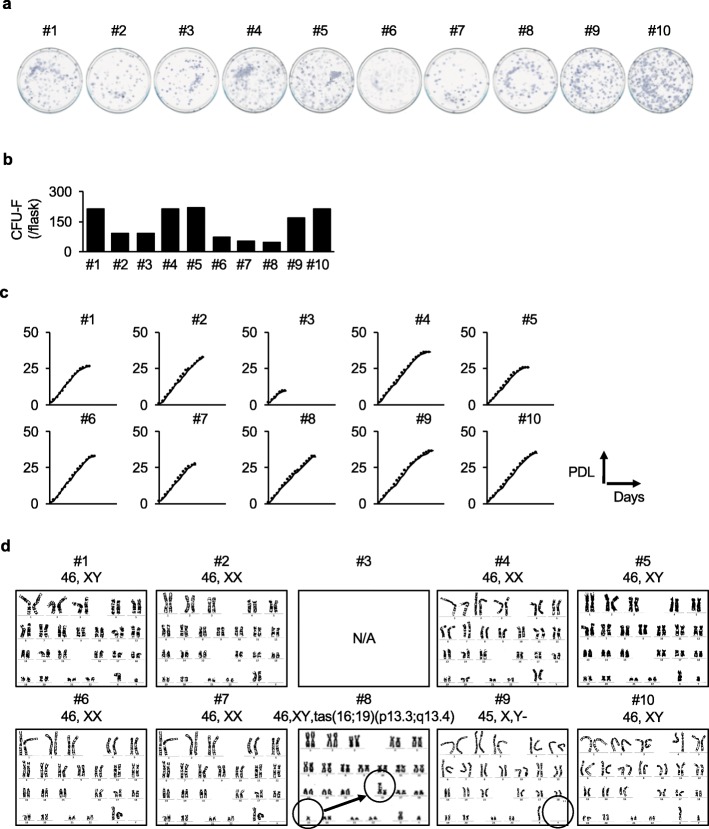
Colony-forming ability, population doubling property, and chromosomal stability of ten donor-derived hDPSCs. **a**, **b** The colony-forming ability of the ten donor-derived hDPSCs was tested by CFU-F assay. Representative images of the CFU-F are shown by toluidine blue staining (**a**). The average number of colonies is shown (**b**). **c** Population doubling assay is analyzed by sequential cell passaging every 7 days. The results are shown as the average total population doubling levels (PDL). PDL were shown at each passage until the ten donor-derived hDPSCs lost their proliferation capacity. **d** Chromosomal stability was tested by G-band karyotype assay in the P10 hDPSC products. Donor #3-derived hDPSCs were excluded because of the very low PDL. A chromosomal aberration was detected in donor #8- and #9-derived P10 hDPSC. N/A, not applicable

We randomly selected ten different healthy pediatric donors, who were clinically diagnosed as healthy without any systemic disease (Table 1). The tooth samples were transport from a clinic to our laboratory at 4 °C within 24 h before we start to isolate hDPSCs. Their hDPSCs were successfully able to form attached colonies containing spindle-shaped cells on the plastic culture flasks (Fig. 2a, b).

**Fig. 3:**
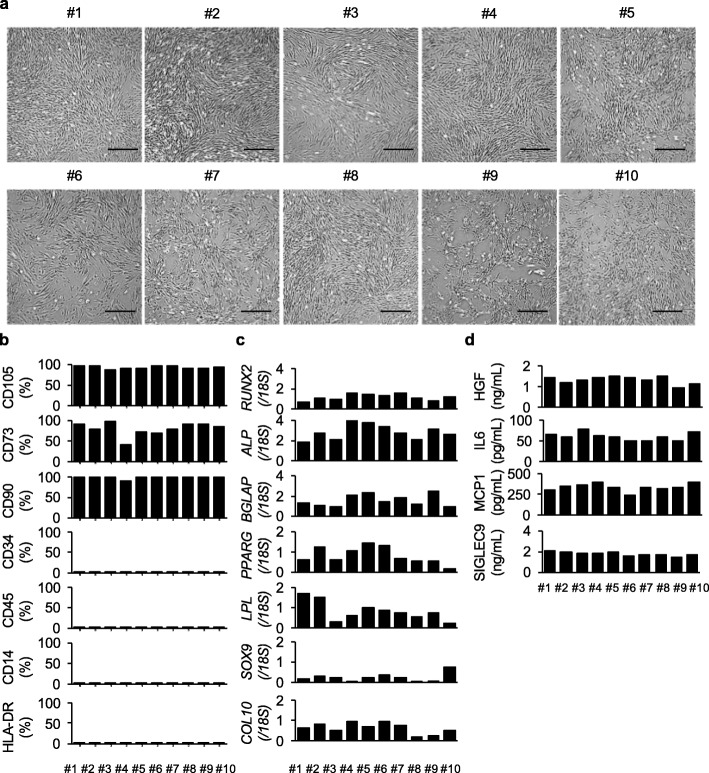
Characterization of the properties of P3 hDPSC products at freezing for the MCB. P3 hDPSC products were independently produced by the passage and expansion of the CFU-F-formed cells from ten donors. Characterization of the properties of the P3 hDPSC products was carried out before MCB storage. **a** Morphology of the P3 hDPSCs is shown as the representative microscopic images of each donor. Bars = 100 μm. **b** Cell surface marker analysis of the P3 hDPSCs was tested by flow cytometric assay. The results are shown as the average positive rates of each marker. **c** Multipotency of the P3 hDPSCs was assessed by the differentiation-specific gene expression of human osteoblasts, adipocytes, and chondrocytes in the P3 hDPSCs 1, 6, and 4 weeks after the induction, respectively, quantitative reverse transcription polymerase chain reaction (qRT-PCR) assay. The results are shown as the ratios of the expression of *18S* and are shown as the average ratio. *ALP*, alkaline phosphatase gene; *BGLAP*, bone gamma-carboxyglutamine protein gene; *COL10*, collagen type X gene; *GAPDH*, glyceraldehyde-3-phosphate dehydrogenase gene; *LPL*, lipoprotein lipase gene; *PPARG2*, peroxisome proliferator activated receptor gamma 2 gene; *RUNX2*, runt-related transcription factor 2 gene; *SOX9*, SRY-box 9 gene. **c** Secretion of anti-inflammatory factors from the P3 hDPSCs was tested by enzyme-labeled immunosorbent assay (ELISA). The results are shown as the average concentration of hepatocyte growth factor (HGF), interleukin 6 (IL6), monocyte chemotactic protein 1 (MCP1), and sialic acid-binding immunoglobulin-type lectin 9 (SIGLEC9) in the conditioned medium of each donor. **b–d** The graph bars show the mean. *n* = 3 for all groups

We produced the P3 hDPSC products from the colony-forming hDPSCs and stored them in liquid nitrogen for the MCB. We then subjected some cryopreserved P3 MCB products to a population doubling assay. The hDPSC products were sequentially passed every 7 days until they lost their proliferative capacity. All ten donor-derived hDPSC products commonly exhibited a limited capacity of cell proliferation (Fig. 2c, Supplementary Table 1). Only donor #3-derived hDPSC products stopped its growth at P7 with a PDL of 9.7. The other 9 donor-derived hDPSC products exceeded a PDL of 26, and the P10 hDPSC products showed a PDL of 20, indicating that the donor #3-derived hDPSC products were excluded to prepare for the WCB batch (Fig. 1).

**Fig. 4:**
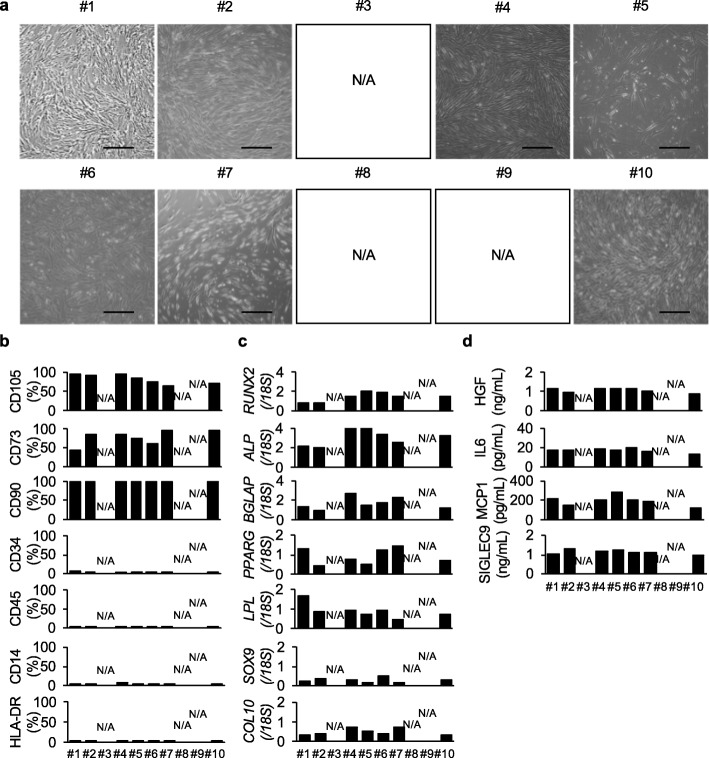
Characterization of the properties of P10 hDPSC products for the WCB. P10 hDPSC products were manufactured from cryopreserved hDPSC products in the MCB. Donor #3-, #8-, and #9-derived hDPSC products were discarded because of the low proliferation and chromosomal aberrations, as shown in Fig. 1. The other seven donor-derived P10 hDPSC products were characterized before WCB cryopreservation. **a** Morphology of the P10 hDPSCs is shown as a representative microscopic image. Bars = 100 μm. **b** Cell surface marker analysis of each donor P10 hDPSCs was tested by flow cytometry. The results are shown as the average positive rates of each marker. **c** Multipotency of the P10 hDPSCs was assessed by the differentiation-specific gene expression of human osteoblasts, adipocytes, and chondrocytes 1, 6, and 4 weeks after the induction, respectively, by qRT-PCR assay. The results are shown as the ratios of the expression of *18S* and are shown as the average ratio. **d** Secretion of anti-inflammatory factors from the P10 hDPSCs was tested by ELISA. The results are shown as the concentration of each factor in each donor. **a–d** N/A, not applicable. **b–d** The graph bars show the mean. *n* = 3 for all groups

In parallel, we tested the chromosomal stability and microorganism safety of the nine donor-derived P10 hDPSC products for the WCB. A chromosomal aberration was detected in the donor #8- and #9-derived P10 hDPSC products, but not in the other six donor-derived P10 hDPSC products (Table 2, Fig. 2d). Pathogenic microorganism contamination was not detected in all P10 hDPSC products (Table 3). All P3 hDPSC products for the MCB also passed both tests (Tables 2 and 3). These findings indicated that donor #3-, #8-, and #9-derived hDPSC products did not pass the present basic criteria; therefore, the other seven donor (donor #1, #2, #4–#7, and #10)-derived-P10 hDPSC products were used for further validation for the WCB.

**Table 2:** Summary of chromosomal safety of human deciduous pulp stem cell products

Donor	P3 (in 20 cells)	P10 (in 20 cells)
#1	46, XY (20 cells)	46, XY (20 cells)
#2	46, XX (20 cells)	46, XX (20 cells)
#3	46, XY (20 cells)	N/A
#4	46, XX (20 cells)	46, XX (20 cells)
#5	46, XY (20 cells)	46, XX (20 cells)
#6	46, XX (20 cells)	46, XX (20 cells)
#7	46, XX (20 cells)	46, XX (20 cells)
#8	46, XX (20 cells)	46, XX (17 cells), 46, XX, tas(16;19) (p13.3;q13.4) (3 cells)
#9	46, XY (20 cells)	46, XY (16 cells), 45, XY- (4 cells)
#10	46, XY (20 cells)	46, XY (20 cells)

P3, passage 3 human deciduous pulp stem cell products; P10, passage 10 human deciduous pulp stem cell product. *N/A* not available

**Table 3:** Summary of microorganism safety of human deciduous pulp stem cell products

Donor	Endotoxin (EU)	FTA-ABS	Mycoplasma	HCV	HBV	HTLV-1	Parbovirus	CMV	HIV-1
**P3**									
#1	0.025	–	–	–	–	–	–	–	–
#2	0.021	–	–	–	–	–	–	–	–
#3	0.023	–	–	–	–	–	–	–	–
#4	0.022	–	–	–	–	–	–	–	–
#5	0.025	–	–	–	–	–	–	–	–
#6	0.027	–	–	–	–	–	–	–	–
#7	0.023	–	–	–	–	–	–	–	–
#8	0.023	–	–	–	–	–	–	–	–
#9	0.022	–	–	–	–	–	–	–	–
#10	0.025	–	–	–	–	–	–	–	–
**P10**									
#1	0.024	–	–	–	–	–	–	–	–
#2	0.023	–	–	–	–	–	–	–	–
#3	N/A	N/A	N/A	N/A	N/A	N/A	N/A	N/A	N/A
#4	0.026	–	–	–	–	–	–	–	–
#5	0.023	–	–	–	–	–	–	–	–
#6	0.025	–	–	–	–	–	–	–	–
#7	0.024	–	–	–	–	–	–	–	–
#8	0.025	–	–	–	–	–	–	–	–
#9	0.021	–	–	–	–	–	–	–	–
#10	0.027	–	–	–	–	–	–	–	–
**Thawed-P10**									
#2	0.023	–	–	–	–	–	–	–	–
#10	0.024	–	–	–	–	–	–	–	–

*P3* passage 3 human deciduous pulp stem cell products, *P10* passage 10 human deciduous pulp stem cell products, *Thawed-P10* Thawed-P10 human deciduous pulp stem cell products, *CMV* cytomegalovirus, *FTA-ABS* fluorescent treponemal antibody absorption test, *HBV* hepatitis B virus, *HCV* hepatitis C virus, *HIV-1* human immunodeficiency virus type 1, *HTLV-1* human T-cell leukemia virus type 1

### Validation of the properties of hDPSC products for the MCB and the WCB

#### Characterization of P3 and P10 hDPSC products

We tested the properties of the ten donor P3 and seven donor (donor #1, #2, #4–#7, and #10)-derived P10 hDPSC products, which passed the present basic criteria, based on four criteria including cell morphology, cell surface marker expression, multi-differentiation potential, and immunosuppressive factor release. All P3 and P10 hDPSC products adhered on plastic culture dishes and showed a typical fibroblast-like morphology (Figs. 3a and 4a). A flow cytometric analysis showed that all P3 and P10 hDPSC products expressed MSC markers including CD105, CD73, and CD90 (Figs. 3b and 4b, Supplementary Figs. 1 and 2, Supplementary Table 2). Meanwhile, both P3 and P10 hDPSC products commonly lacked the expression of hematopoietic cell markers including CD34, CD45, and CD14, and HLA class II HLA-DR (Figs. 3b and 4b, Supplementary Figs. 1, 2, Supplementary Table 2). A multi-differentiation test demonstrated that the both passage products were capable of differentiating into the typical mesenchymal 3-lineage cells, as indicated by the expression of lineage-specific genes for osteoblasts (*RUNX2*, *ALP*, and *BGLAP*), chondrocytes (*SOX9*, *COL2*, and *COL10*), and adipocytes (*PPARG* and *LPL*) by qRT-PCR assay (Figs. 3c and 4c). An ELISA showed the ability to secrete anti-inflammatory factors including HGF, IL6, MCP1, and SIGLEC9 in the CM of theP3 and P10 hDPSC products (Figs. 3d and 4d). Our results presented no significant difference as MSCs including hDPSCs between P3 and P10 hDPSC products from donor #1, #2, #4–#10, indicating that donor #1, #2, #4–#10-derived-P10 hDPSC products maintain the minimum requirement to establish working cell bank for MSCs including hDPSCs.

#### Tumorigenicity and immunogenicity of P10 hDPSC products

**Fig. 5:**
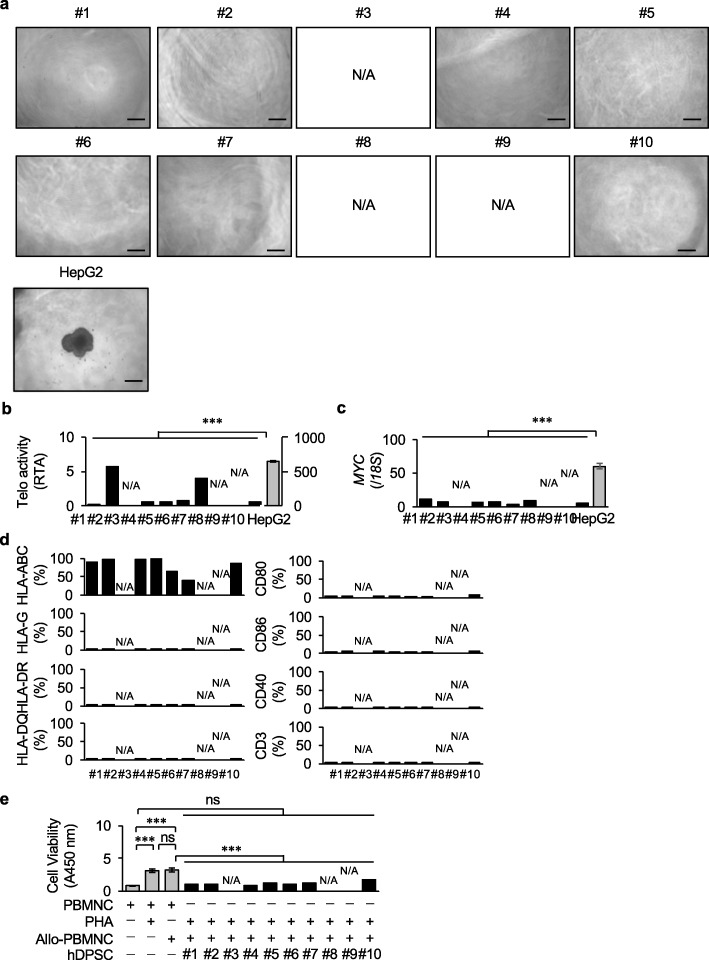
Tumorigenesis and immunogenicity of P10 hDPSC products. **a** Tumorigenicity of the P10 hDPSCs was performed by colony-forming assay in soft agar. The results are shown as representative microscopic images 12 weeks after culture. **b** Telomerase (Telo) activity in the P10 hDPSCs was analyzed by the telomeric repeat amplification protocol. The results are shown as the average activity of relative telomerase activity (RTA). **c** Expression of *MYC* in the P10 hDPSC products was analyzed by qRT-PCR. The results are calculated as the ratios of the expression of 18S ribosomal RNA (*18S*) and are shown as the average ratio. **d** Immunogenic antigens in the P10 hDPSCs were analyzed by flow cytometry. The results are shown as the average positive rates of each marker. HLA, human leukocyte antigen. **e** The in vitro immunogenic reaction of the P10 hDPSCs was analyzed using a mixed lymphocyte reaction test. Peripheral blood mononuclear cells (PBMNCs) were mixed with gamma-irradiated hDPSCs and allogenic PBMNCs (Allo-PBMNCs), and its viability was measured at 450 nm (A450 nm). Phytohemagglutinin (PHA) was used as a T cell mitogen. The results are shown as the average viability. **a–e** N/A, not applicable. **a–c** HepG2, HepG2 cells are used as a positive control. **b–e***n* = 3 for all groups. The graph bars show the mean (black columns) or mean ± SEM (gray columns). ***P < 0.005. ns, not significant

We additionally investigated the tumorigenicity and immunogenicity of the seven donor-derived P10 hDPSC products for the WCB, which passed both the basic and MSC criteria. A colony formation assay with a soft agar culture system indicated that all P10 cell products were not able to form cell clusters compared to the human liver cancer cell line HepG2 (Fig. 5a). Telomerase activity and qRT-PCR assays revealed that all P10 hDPSC products expressed significantly low levels of telomerase activity and *MYC* in comparison with HepG2 cells (Fig. 5b, c). A flow cytometric analysis showed that all seven donor-derived P10 hDPSC products lacked the expression of HLA class II antigens HLA-DR and HLA-DQ, T-cell cofactors CD80, CD86, and CD40, and T-cell marker CD3 (Figs. 5d, Supplementary Fig. 3). While, the intact P10-hDPSC products expressed HLA class I HLA-ABC, but not the class I antigen HLA-G (Figs. 5d, Supplementary Fig. 3). A mixed lymphocyte culture test determined that all P10 hDPSC products did not stimulate the cell viability of phytohemagglutinin activated allogenic PBMNCs (Fig. 5e).

#### Preclinical evaluation of P10 hDPSC products

**Fig. 6:**
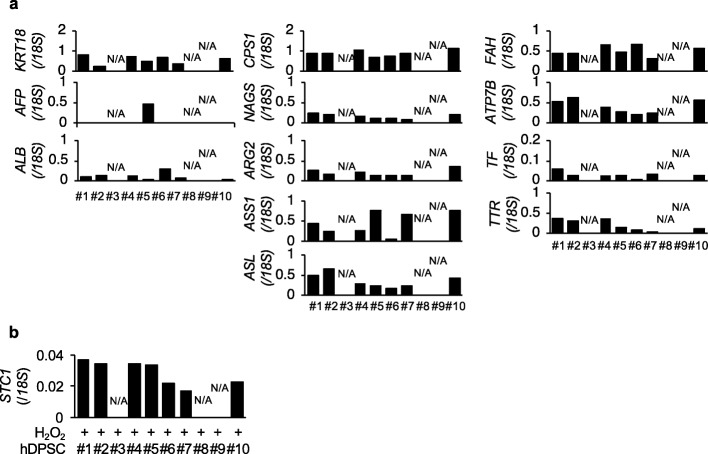
Hepatogenic capacity of P10 hDPSC products for the WCB. **a** The hepatogenic capacity of the P10 hDPSCs was tested by hepatocyte-specific gene expression 4 weeks after hepatogenic inductive culture by qRT-PCR assay. The results are shown as the ratios of the expression of *18S* and are shown as the average ratio. *AFP*, alpha fetoprotein gene; *ALB*, albumin gene; *ARG2*, arginase 2 gene; *ASL*, argininosuccinate lyase gene; *ASS1*, argininosuccinate synthase 1 gene; *ATP7B*, ATPase copper transporting beta gene; *CPS1*, carbamoyl-phosphate synthase 1 gene; *CYP3A7*, cytochrome P450 family 3 subfamily A member 7 gene; *FAH*, fumarylacetoacetate hydrolase gene; *KRT18*, keratin 18 gene; *NAGS*, *N*-acetylglutamate synthase gene; *TF*, transferrin gene; *TTR*, *transthyretin*. N/A, not applicable. The graph bars show the mean. **b** The P10 hDPSCs were stimulated with and without H_2_O_2_ to analyze the expression of stanniocalcin 1 (*STC1*) by qRT-PCR. The results are calculated as the ratios of the expression of *18S* and are shown as the average ratio. **a**, **b***n* = 3 for all groups. N/A, not applicable

An in vivo therapeutic efficacy test for chronic liver disease was conducted in the seven donor-derived P10 hDPSC products. The seven-donor-derived P10 hDPSC products under a hepatogenic culture protocol showed differentiation into hepatocyte-like cells, as indicated by expression of hepatocyte-specific genes by qRT-PCR assay (Fig. 6a). STC1 was secreted from MSCs during anti-oxidative stress in vitro and during an anti-fibrotic response in vivo in CCl_4_-injured mouse liver tissues [25]. The P10 hDPSC products expressed a marked expression of *STC1* under hydrogen peroxide treatment (Fig. 6b), indicating that P10 hDPSC products might be a clinically promising product to regenerate damaged liver.

**Fig. 7:**
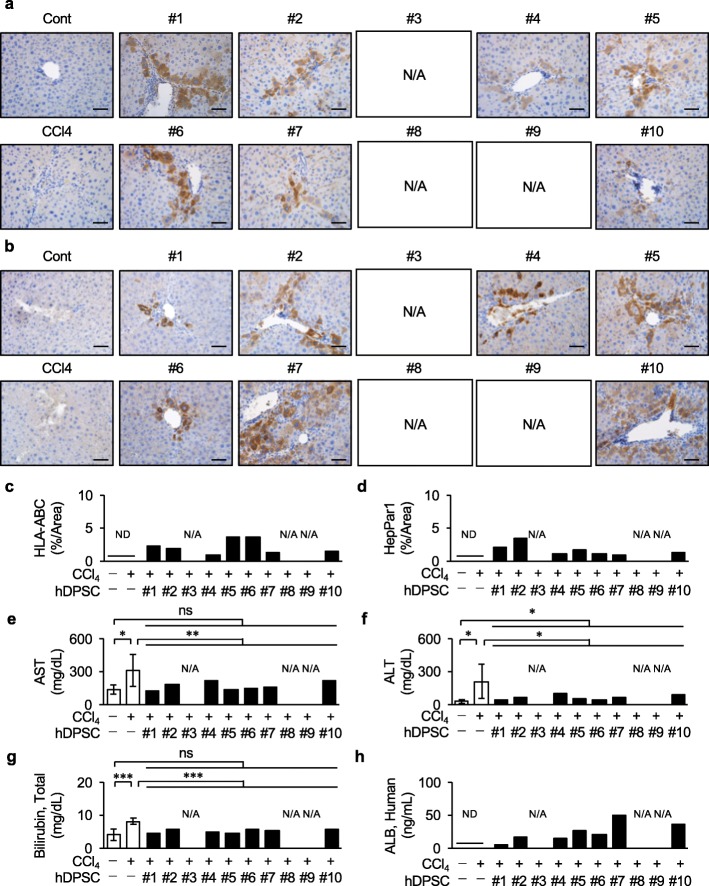
Transplantation of P10 hDPSC products improved chronically CCl_4_-damaged liver dysfunction in mice. Each donor-derived P10 hDPSC product was intrasplenically transplanted into 4-week-CCl_4_-treated immunocompetent mice without immunosuppressant (*n* = 5). The age-matched control non-CCl_4_-treated (*n* = 5) and non-transplanted CCl_4_-treated (*n* = 5) immunocompetent mice were used as experimental controls. **a–d** Distribution of the donor cells in the CCl_4_-damaged fibrotic liver tissues was investigated by immunohistochemical assay. The results are shown as the representative microscopic images using anti-HLA-ABC (**a**) and anti-hepatocyte paraffin 1 antigen (HepPar1) (**b**) antibodies. Nuclei were stained with hematoxylin. Bars = 50 μm (**a, b**). The results are shown as the ratios of the HLA-ABC- (**c**) and HepPar1- (**d**) positive areas in the mouse liver tissue. **e–h** Serum levels of aspartate aminotransferase (AST, **e**), alanine aminotransferase (ALT, **f**), total bilirubin (**g**), and human albumin (ALB, **h**) were examined by biochemical assays and ELISA. **a–h** N/A, not applicable. **c–h***n* = 5 for all groups. *P < 0.05, ***P < 0.005. The graph bars show the mean ± SEM (white columns) or the mean (black columns). ND, not detected; ns, not significant

**Fig. 8:**
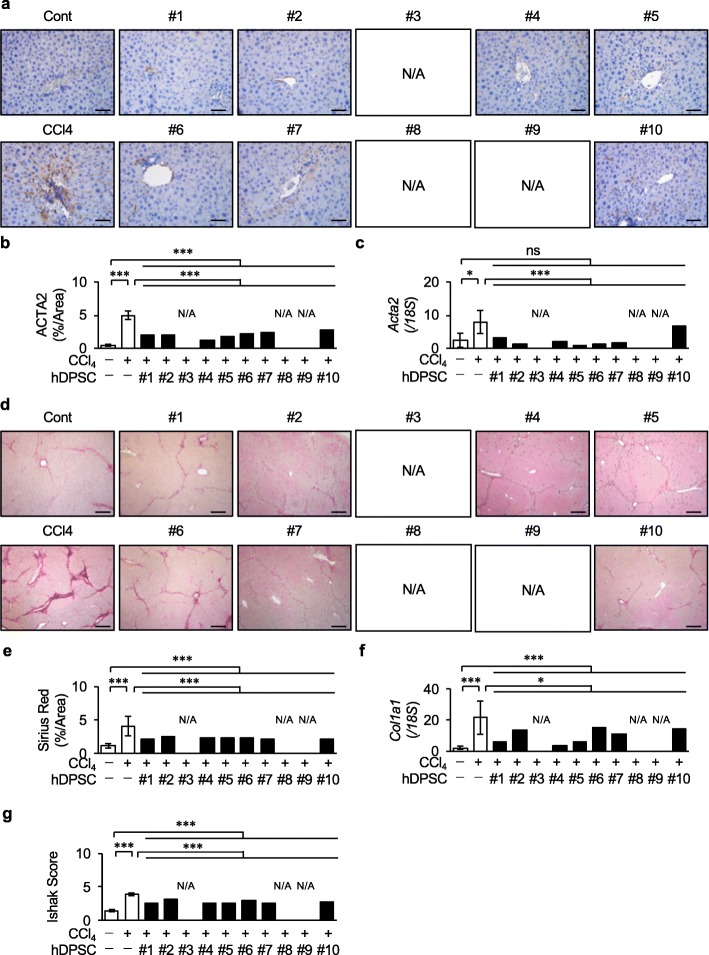
Transplantation of P10 hDPSC products improves CCl_4_-induced liver fibrosis in mice. Each donor-derived P10 hDPSC products were intrasplenically transplanted into 4-week-CCl_4_-treated immunocompetent mice without immunosuppressants (*n* = 5). The age-matched control non-CCl_4_-treated (*n* = 5) and non-transplanted CCl_4_-treated (*n* = 5) immunocompetent mice were used as experimental controls. **a–c** Expression of the activated stellate cells in the CCl_4_-damaged fibrotic liver tissues was investigated. Representative immunohistochemical images show the distribution of actin alpha 2 smooth muscle, aorta (ACTA2) in mouse liver tissue. Nuclei were stained with hematoxylin. Bars = 50 μm (**a**). The area of ACTA2-positive cells in mouse liver tissue was analyzed (**b**). The expression of the *Acta2* was investigated by qRT-PCR (**c**). **d–f** Deposition of fibrous tissues in CCl_4_-damaged liver tissues was investigated. Representative histological images show the distribution of fibrous tissue in mouse liver tissue by picrosirius red staining. Bars = 50 μm (**d**). The picrosirius red-positive area in mouse liver tissue was analyzed (**e**). The expression of the collagen type I alpha 1 chain gene (*Col1a1*) was investigated by qRT-PCR. The results are shown as the ratios of the expression of *18S* (**f**). The liver fibrosis stages were assessed by Ishak scoring (**g**). **a–g** N/A, not applicable. **b**, **c**, **e–g***n* = 5 for all groups. *P < 0.05, ***P < 0.005. The graph bars show the mean ± SEM (white columns) or the mean (black columns). ns, not significant

Prior to the validation of P10 hDPSC products, we examined therapeutic effects of the P10 hDPSCs-products in chronically CCl_4_-injured immunocompetent BL/6 mice. We evaluated that all donor-derived P3 hDPSCs-products recovered liver function in recipient chronically CCl_4_-injured BL/6 mice as indicated by the significant reduction of AST, ALT, and total bilirubin in the serum detection of human albumin in the serum (Supplementary Fig. 4). Next, the P10 hDPSC products were transplanted into chronically CCl_4_-injured immunocompetent BL/6 mice via the spleen without any immunosuppressant before or after the transplantation. An immunohistochemical assay using anti-HLA-ABC and anti- HepPar1 antibodies confirmed direct liver regeneration by the in situ transformation of the transplanted P10 hDPSC products (Fig. 7a–d). Biochemical assays revealed that the P10 hDPSC transplantation recovered damaged liver functions as indicated by a significant reduction of AST, ALT, and total bilirubin in the serum of the P10 hDPSC-transplanted group (Fig. 7e–g) and detection of human albumin in the serum of the P10 hDPSC-transplanted group (Fig. 7h). Interestingly, there was no significant difference in the effects on AST, ALT, total bilirubin, and human albumin in serum between P3 and P10 hDPSC products in the present chronic liver fibrosis. The P10 hDPSC transplantation decreased the activation of hepatic stellate cells as indicated by the decreased immunoreactivity for ACTA2 and the reduced gene expression of *Acta2* in the CCl_4_-induced fibrous liver tissue by qRT-PCR and immunohistochemical analyses (Fig. 8a–c). The P10 hDPSC transplantation improved CCl_4_-induced liver fibrosis as indicated by the markedly suppressed area of fibrous tissue-deposition and the degree and expression of *Col1a1* in the P10 hDPSC-transplanted group by Picrosirius Red staining, Ishak scoring, and qRT-PCR (Fig. 8d–g). Moreover, we further found that the P10 hDPSC transplantation reduced liver fibrotic-related genes and proteins including metalloproteinases 2 (Mmp2), Mmp3, inhibitor of metalloproteinase 1 (Timp1), Timp2, and transforming growth factor beta in the CCl_4_-induced mouse fibrous liver tissue by qRT-PCR analysis and ELISA (Supplementary Fig. 5). These findings suggested that the P10 hDPSC products were a feasible therapeutic option for chronic liver fibrosis.

### Validation of final hDPSC products for chronic liver fibrosis treatment

**Fig. 9:**
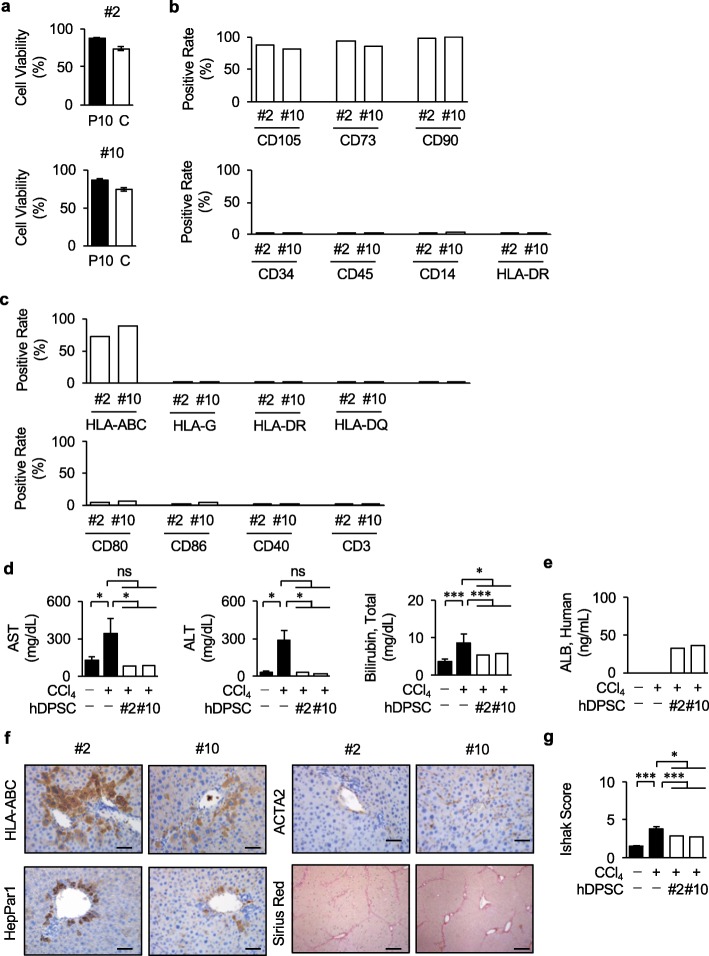
Transplantation of final hDPSC products from WCB improves CCl_4_-induced pro-fibrotic markers in mice. Two cryopreserved hDPSC products in WCB (donor #2 and #10) were randomly selected from the WCB batch and were thawed to use as final hDPSC products for the quality check. **a** Cell viability of the final hDPSC products. The final hDPSC products thawed from 10 vials. They were stained with trypan blue, and living cells were counted. Black columns, living cell numbers of the final hDPSC products at freezing; White columns, living cell number of the final hDPSC products after thawing. The graph bars show the mean ± SEM. *n* = 10. **b, c** Cell surface marker analysis of the final hDPSC products was tested by flow cytometric assay. Markers for mesenchymal stem cells (**b**) and immunogenic antigen (**c**) were assessed in the final hDPSC products. The results were shown as the averaged positive rates of each marker in each donor. *n* = 3. **d–f** Each final hDPSC products were intrasplenically transplanted into 4-week-CCl_4_-treated immunocompetent mice without immunosuppressants (*n* = 5). The age-matched control non-CCl_4_-treated (*n* = 5) and non-transplanted CCl_4_-treated (n = 5) immunocompetent mice were used as experimental controls. Serum levels of AST, ALT, total bilirubin (**d**), and human ALB (**e**) were examined by biochemical assays and ELISA. Distribution of the donor cells and deposition of fibrous tissues in the CCl_4_-damaged fibrotic liver tissues was investigated by immunohistochemical assay and collagen staining. The results are shown as representative images stained with antibodies against HLA-ABC, HepPar1, ACTA2, and Picrosirius Red. Nuclei are stained with hematoxylin. Bars = 50 μm (**f**). The liver fibrosis stages were assessed by Ishak scoring (**g**). **d, e, h***n* = 5 for all groups. *P < 0.05, ***P < 0.005. The graph bars show the mean ± SEM (black columns) or the mean (white columns). ns, not significant

Colony number is a relatively easy and early evaluation means in the cell manufacturing process. In this study, we divided donors into two groups; donors #2, #3, #6, #7, and #8 were a low colony-forming ability group, and the other donors #1, #4, #5, #9, and #10 were a high colony-forming ability group. We then randomly selected donors #2 and #10 from each group and used as the final hDPSC products for clinical validation to assess whether colony-forming ability of donors can determine the therapeutic efficacy of hDPSCs. The mean viability of the two donor-derived final hDPSC products after thawing was 73.4 ± 4.5% and 69.8 ± 7.2%, respectively, while the pre-cyropreserved viability was 91.4 ± 3.9% and 89.8 ± 4.1%, respectively (Fig. 9a). From the results of cell surface antigen expression by flow cytometric analysis, the two clinical hDPSC products were evaluated for their MSC immunophenotype and less immunogenicity (Fig. 9b, c, Supplementary Fig. 6, Supplementary Tables 2, 3). Microorganism tests confirmed no contamination with pathogens (Table 3). Furthermore, transplantation of the two final hDPSC products evaluated the therapeutic efficacy of liver regeneration via direct in situ transformation and anti-fibrotic effects in a CCl_4_-induced mouse model of liver fibrosis by biochemical and immunological serum assays, immunohistochemical analyses, Picrosirius Red staining, and Ishak scoring (Fig. 9d–g, Supplementary Fig. 7). However, there was no significant therapeutic difference between the two final products.

## Discussion

In this study, we established a SOP for manufacturing clinical-grade hDPSC products from CFU-F-derived multi-colonies under XF/SF condition and built a two-step cryopreservation system including an MCB and a WCB in the procedure. The MCB was the first storage step for hDPSC products with high proliferation potential. The hDPSC products cryopreserved in the MCB were passaged and expanded to storage in the WCB, which would directly supply large numbers of the final hDPSC products to patients off-the-shelf. We then employed basic criteria including a PDL of 20, chromosomal stability, and microorganism safety, to manufacture enough numbers of safety products for the WCB. In the present study, we tested ten donor-derived hDPSC products whether each product meets the basic criteria. Finally, the seven donor-derived hDPSC products for the WCB products were obtained. The three donor-derived hDPSC products did not meet our basic criteria; one donor-derived hDPSC product showed very low population doubling ability, and the others showed chromosomal abnormalities in the WCB. All seven donor-derived hDPSC products fulfilled the stem cell properties including morphology, imuunophenotype, multipotency, and immunosuppressive function. They also showed the preclinical criteria including low immunogenicity, less tumorigenicity, hepatogenic capacity, and in vivo therapeutic efficacy in a liver fibrosis mouse model. Moreover, randomly selected two donor-derived final hDPSC products were verified the clinical safety and applicability for treating chronic liver fibrosis, suggesting that the present SOP, for the first time, provides a manufacturing of clinical-grade hDPSC products for treating chronic liver fibrosis.

Heterogeneous serum such as fetal bovine serum (FBS) has been generally used as an essential source of growth factors and other components to isolate and expand hDPSCs in vitro [21, 22, 26]. The use of FBS involves many safety concerns for clinical application. FBS potentially contains pathogens such as mycoplasmas, prions, or viruses, resulting in immune/allergic reactions to the host. Animal-derived proteins and peptides in FBC are internalized into MSCs during in vitro expansion, resulting in the rejection of donor cells [30–32]. Substantial batch-to-batch variation of FBS is also a disadvantage to the large quantities [31, 33]. Meanwhile, homogeneous serum including human serum and human platelet lysate is proposed as an alternative to FBS and has demonstrated efficient support of cell proliferation of MSCs [34, 35]. The significant variation and limited supply of them may indicate the unsuitability in a large-scale manufacturing of MSCs [36]. XF/SF materials have been known to be more practical in the large-scale manufacturing of MSCs without the aforementioned safety concerns [35, 37]. Therefore, the present findings indicate that the present established XF/SF SOP might be a practical and robust procedure for manufacturing large amounts of clinical-grade hDPSC products.

Several passages, long-term expansion, and large-scale banking are required to manufacture enough numbers of clinical-grade hDPSC products. Recent clinical trials including a Phase II/III trial for acute graft versus host disease (GVHD) and a Phase I/II trial for pediatric metabolic liver diseases show approximately 1–10 × 10^9^ cells per patient as the prospected clinical dose of MSCs [38, 39]. Since we can obtain at least 1 × 10^6^ cells from one deciduous tooth (generally 2–3 × 10^6^ cells from one deciduous tooth) from CFU-F colony-forming P0 cells with a doubling capacity of PDL 20, we could prepare approximately 10,000,000 WCB units containing 1 × 10^6^ clinical-grade hDPSC products per unit, resulting in a therapeutic option for 1000–10,000 patients. Therefore, the present PDL threshold 20 in clinical-grade hDPSC products is a reasonable criterion to secure the supply of the hDPSC products to patients.

Clinical-grade human MSC products should meet critical requirements including normal genetic karyotype and chromosomal stability during long-term culturing and after cryopreserved cell banking [40, 41]. Human MSCs have been considered to be relatively genetically stable during culturing in vitro [42–48]. On the contrary, abundant recent studies have revealed that human MSCs exhibited spontaneous genomic alternation with increased passage number [49–54]. Previous chromosomal profiling studies also provide that karyotypical abnormalities and chromosomal instability in early passaged human MSCs disappear during the extended passage [51, 54–56]. A study further reports that ten batched clinical-grade human MSC products not only occur 20% of non-random chromosomal abnormality, but also recur the abnormality in multiple products from the same batch [57]. In this study, we revealed karyotype abnormality in two hDPSC products for the WCB, but not for the MCB. Meanwhile, the abnormal hDPSC products for the WCB exhibited no tumorigenicity and showed no difference of the stem cell properties and therapeutic efficacy when compared to the normal hDPSC products for the WCB (data not shown). Heterogeneity of dental stem cells plays a crucial role in the genetic stability to suppress tumorigenesis and differentiation capacity [58]. These findings suggest that the quality and type of chromosomal abnormality may occur in hDPSC products in a donor-dependent manner in an ex vivo cell processing. Further studies will be necessary to evaluate the significance and mechanism of chromosomal alternations and their associating therapeutic safety of hDPSC products.

Long-term manufacturing of clinical-grade MSCs in vitro may incur chromosomal aberrations and microorganism concerns [59, 60], indicating that the preliminary sorting of chromosomal stability and microorganism contamination in hDPSC products for the MCB and the WCB is essential and critical safety steps required for obtaining clinical applications the final hDPSC products. The present microorganism tests in hDPSC products are a reasonable verification of microorganism safety. The present chromosomal safety was validated only with G-band karyotyping in hDPSC products. G-band karyotyping is a classical, useful, and short chromosomal analysis, but fails to detect cryptic rearrangements or aberrations covering small areas, indicating that additional chromosomal tests are required. A parallel chromosomal assay using advanced cytogenetic techniques such as spectral karyotyping by fluorescent in situ hybridization and comparative genomic hybridization array may supply a more sophisticated evaluation of genetic stability in clinical-grade hDPSC products [54].

The present manufacturing and quality tests indicate the safe and sufficient production of clinical-grade hDPSC products for treating chronic liver fibrosis. However, several difficulties remain to meet good manufacturing practice (GMP) in the present procedure for clinical-grade hDPSC products. In particular, the present study lacked in vivo toxicological validation of clinical-grade hDPSC products. The further toxicity test should be designed to validate the quality and reproducibility regarding the safety of clinical-grade MSC products in animals to support the safety claim in patients.

## Conclusion

In this study, we established a XF/SF-conditioned procedure for the clinical-grade production of hDPSCs from multiple CFU-F colonies. We built in a two-step cell banking system, the MCB and the WCB, and obtained final hDPSC products. Because deciduous dental pulp tissue is an accessible and practical stem cell source for research and therapy, the present manufacture, validation, characterization, and preclinical results indicate that the present manufacturing procedure will provide clinically feasible hDPSC products to treat chronic liver fibrosis.

## Supplementary information

Additional file 1: Supplementary Methods. Table S1. Summary of population doubling level in human deciduous pulp stem cell (hDPSC) products. Table S2. Summary of expression of cell surface antigens for mesenchymal stem cells in human deciduous pulp stem cell products. Table S3. Summary of expression of cell surface antigens for immunogenicity in human deciduous pulp stem cell products. Table S4. Antibody information for flow cytometry. Table S5. TaqMan probe information for qRT-PCR assay to human adipocyte-, chondrocyte-, and osteoblast-specific genes. Table S6. TaqMan probe information for qRT-PCR assay to human hepatocyte-specific genes. Table S7. TaqMan probe information for real-time RT-PCR. Table S8. Commercially available kit information for biochemical analysis and enzyme-labeled immunosorbent assay. Table S9. Antibody information for immunohistochemistry. Fig. S1. Cell surface marker analysis of the P3 hDPSCs was tested by flow cytometric assay. The results are shown as representative histograms. The red- and black-colored histograms indicate the frequency stained with target antigen-specific and isotype-matched antibodies, respectively. R-PE, Rphycoerythrin. Fig. S2. Cell surface marker analysis of the P10 hDPSCs was tested by flow cytometric assay. The results are shown as representative histograms. The red- and black-colored histograms indicate the frequency stained with target antigen-specific and isotype-matched antibodies, respectively. R-PE, Rphycoerythrin. Fig. S3. Cell surface marker analysis of the P10 hDPSCs was tested by flow cytometric assay. The results are shown as representative histograms. The red- and black-colored histograms indicate the frequency stained with target antigen-specific and isotype-matched antibodies, respectively. R-PE, Rphycoerythrin. Fig. S4. Transplantation of P3 hDPSC products improved chronically CCl4-damaged liver dysfunction in mice. Fig. S5. Anti-fibrotic effects of P10 MCB-hDPSC transplantation in mouse CCl4-induced fibrotic liver. Fig. S6. Cell surface marker analysis of the P10 hDPSCs was tested by flow cytometric assay. Markers for mesenchymal stem cells (a) and immunogenic antigen (b) were assessed in the final hDPSC products. The results are shown as representative histograms. The red- and black-colored histograms indicate the frequency stained with target antigen-specific and isotype-matched antibodies, respectively. R-PE, R-phycoerythrin. Fig. S7. Transplantation of final hDPSC-products from WCB improves CCl4-induced pro-fibrotic markers in mice. (PDF 5848 kb)

## Data Availability

All data generated or analyzed during this study are included in this published article and its supplementary information files.

## Abbreviations

ACTA2: Actin, alpha 2, smooth muscle
*Acta2*: Actin, alpha 2, smooth muscle gene
ALT: Alanine aminotransferase
AST: Aspartate transferase
CFU-F: Colony-forming unit-fibroblast
CM: Conditioned medium
CMV: Cytomegalovirus
*Col1a1*: Collagen type I alpha 1a gene
ELISA: Enzyme-linked immunosorbent assay
FBS: Fetal bovine serum
FCM: Flow cytometry
GMP: Good manufacturing practice
GVHD: Graft-versus-host disease
HBV: Hepatitis B virus
HCV: Hepatitis C virus
hDPSCs: Human deciduous pulp stem cells
HepPar1: Human hepatocyte antigen paraffin 1
HGF: Hepatocyte growth factor
HIV-1: Human immunodeficiency virus 1
HLA-ABC: Human leukocyte antigen A, B, and C
HLA-DQ: Human leukocyte antigen DQ
HLA-DR: Human leukocyte antigen DR
HLA-E: Human leukocyte antigen E
HTLV-1: Human T-cell leukemia virus type 1
IL6: Interleukin 6
MCB: Master cell bank
MCP1: Monocyte chemotactic protein 1
*Mmp2*: Metalloproteinases 2 gene
*Mmp3*: Metalloproteinases 3 gene
MSCs: Mesenchymal stem cells
OLT: Orthotopic liver transplantation
P1: Passage 1
PBS: Phosphate-buffered saline
PDL: Population doubling level
SD: Standard deviation
SEM: Standard error of the mean
SF: Serum-free
SIGLEC9: Sialic acid-binding immunoglobulin-type lectin 9
SOP: Standard operating procedure
STC1: Stanniocalcin 1
*Tgfb*: Transforming growth factor beta gene
*Timp1*: Tissue inhibitor of metalloproteinase 1 gene
*Timp2*: Tissue inhibitor of metalloproteinase 2 gene
WCB: Working cell bank
XF: Xenogeneic-free
XFM: Xenogeneic-free medium
