# Supplementary material for: A model study for the manufacture and validation of clinical-grade deciduous dental pulp stem cells for chronic liver fibrosis treatment
Source: Stem Cell Res Ther. 2020 Mar 25;11:134. doi: 10.1186/s13287-020-01630-w (PMC7093986; doi:10.1186/s13287-020-01630-w)
Supplement: Supplementary file 1 — Supplementary Methods. Table S1. Summary of population doubling level in human deciduous pulp stem cell (hDPSC) products. Table S2. Summary of expression of cell surface antigens for mesenchymal stem cells in human deciduous pulp stem cell products. Table S3. Summary of expression of cell surface antigens for immunogenicity in human deciduous pulp stem cell products. Table S4. Antibody information for flow cytometry. Table S5. TaqMan probe information for qRT-PCR assay to human adipocyte-, chondrocyte-, and osteoblast-specific genes. Table S6. TaqMan probe information for qRT-PCR assay to human hepatocyte-specific genes. Table S7. TaqMan probe information for real-time RT-PCR. Table S8. Commercially available kit information for biochemical analysis and enzyme-labeled immunosorbent assay. Table S9. Antibody information for immunohistochemistry. Fig. S1. Cell surface marker analysis of the P3 hDPSCs was tested by flow cytometric assay. The results are shown as representative histograms. The red- and black-colored histograms indicate the frequency stained with target antigen-specific and isotype-matched antibodies, respectively. R-PE, Rphycoerythrin. Fig. S2. Cell surface marker analysis of the P10 hDPSCs was tested by flow cytometric assay. The results are shown as representative histograms. The red- and black-colored histograms indicate the frequency stained with target antigen-specific and isotype-matched antibodies, respectively. R-PE, Rphycoerythrin. Fig. S3. Cell surface marker analysis of the P10 hDPSCs was tested by flow cytometric assay. The results are shown as representative histograms. The red- and black-colored histograms indicate the frequency stained with target antigen-specific and isotype-matched antibodies, respectively. R-PE, Rphycoerythrin. Fig. S4. Transplantation of P3 hDPSC products improved chronically CCl4-damaged liver dysfunction in mice. Fig. S5. Anti-fibrotic effects of P10 MCB-hDPSC transplantation in mouse CCl4-induced fibrotic liver. Fi [file 13287_2020_1630_MOESM1_ESM.pdf]

A model of the manufacture and validation of clinical-grade deciduous dental pulp stem cells for chronic liver fibrosis treatment

Tsuyoshi Iwanaka, Takayoshi Yamaza, Soichiro Sonoda, Koichiro Yoshimaru, Toshiharu Matsuura, Sara Murata, Haruyoshi Yamaza, Shouichi Ohga, Yoshinao Oda, and Tomoaki Taguchi

Supplementary Methods

Collection and preparation of conditioned medium (CM) from human deciduous pulp stem cells (hDPSC) product culture

The hDPSC-products at 60–70% confluency were washed with 1 mL of phosphate buffered saline (PBS; Nacalai Tesque, Kyoto Japan) twice, and were cultured for 48 hours with 10 mL of a serum-free (SF) Dulbecco's Modified Eagle Medium without phenol red and antibiotics (DMEM (–); Thermo Fisher Scientific, Waltham, MA). The medium was collected as the CM into a 50 mL polystyrene tube (Corning, Corning, NY), and centrifuged for 3 minutes at 4 ºC and 440 × *g*. The supernatants were discarded into another 50 mL polystyrene tube (Corning), and were centrifuged for 3 minutes at 4 ºC and 17,400 × *g*. Total protein concentration in the CM was measured using a BCA protein assay (Thermo Fisher Scientific) according to the manufacturer's instruction, and was adjusted to 1 mg/mL with SF-DMEM (–).

Hepatogenic induction of hDPSC-products

The hDPSC-products (0.25 × 10^6 per dish) were seeded on human fibronectin-coated 100-mm culture dishes (Corning), and were cultured with 10 mL of a xeno-free medium MSC NutriStem® XF Medium (XFM; Biological Industries, Beit HaEmek, Israel) without antibiotics. After they reached confluency, the hDPSC-products were cultured in a sequential hepatogenic medium based on Iscove's Modified Dulbecco's Media (Thermo Fisher Scientific). The hDPSC-products were preincubated with human epidermal growth factor (20 ng/mL; PeproTech, Rocky Hill, NJ) and human fibroblast growth factor 2 (10 ng/mL; FGF2; PeproTech) for 2 days. They were then stimulated with FGF2 (10 ng/mL; PeproTech), hepatocyte growth factor (20 ng/mL; HGF; PeproTech), and nicotinamide (5 mmol/L; Merck, Darmstadt, Germany) for 7 days, and were finally cultured with oncostatin M (20 ng/mL; PeproTech), dexamethasone (1 μmol/L; Merck), and ITS+ premix (50 mg/mL; Thermo Fisher Scientific) for 21 days. The hDPSC-products were maintained at 37 ºC with 5% CO_2 in a Forma™ CO_2 incubator (Thermo Fisher Scientific). The medium was changed twice weekly. Expression of hepatocyte-specific genes was assessed by reverse transcription polymerase chain reaction (RT-PCR) assay to determine the hepatogenic differentiation

of hDPSC-products. The human hepatoma-cell line HepG2 cells (Riken BRC, Tsukuba, Japan) were used as the positive control.

Telomerase activity assay

Telomerase activity in the hDPSC-products was tested by telomerase repeat amplification protocol (TRAP) using a Telo *TAGGG* Telomerase PCR ELISA^{PLUS} (Merck) according to the manufacturer's instruction. Briefly, the TRAP was performed with telomeric repeats (TTAGGG; Merck) by PCR on a T-100^{TM} thermal cycler (Bio-Rad Laboratories, Hercules, CA). The PCR products were hybridized to digoxigenin (DIG)-labeled detection probes (Merck), and were reacted with horseradish peroxidase-labeled anti-DIG antibodies (Merck). The results were obtained on a Multiskan^{TM} GO microplate spectrophotometer (Thermo Fisher Scientific). HepG2 cells were used as a positive control. Some extracts from the samples were heated at 85 ºC for 10 min, and were used as a negative control. Internal standards and control templates were provided in the kit. The relative telomerase activities within the different samples were calculated according to the manufacturer's instruction.

T cell activity assay

Human peripheral blood mononuclear cells (PBMNCs) were obtained from peripheral blood from non-related donors (25-28 years old) to the pediatric donors for hDPSCs, and were separated by gradient centrifugation using a Ficoll-Paque PLUS density gradient reagent (GE Healthcare Life Sciences, Pittsburgh, PA). Intact PBMNCs (1×10^6 per well) were co-cultured with gamma-irradiated hDPSCs (10×10^3 per well) or gamma-irradiated PBMNCs (100×10^3 per well) in 96-well multiplates (Corning) with a complete medium. The hDPSCs and PBMNCs were treated at a dose of 30 Gy with an MBR-1520R-3 X-ray generator (Hitachi, Tokyo, Japan). The complete medium consisted of RPMI-1640 medium (Merck) with 10% heat-inactivated fetal bovine serum (FBS; Equitech-Bio, Kerrville, TX), L-glutamine (2 mmol/L; Nacalai Tesque), sodium pyruvate (1 mmol/L; Nacalai Tesque), and premixed antibiotics (100 U/ml penicillin and 100 µg/mL streptomycin; Nacalai Tesque). Some intact PBMNCs (1×10^6 per well) were pre-treated for 24 hours with the T-cell mitogen phytohemagglutinin (PHA; 5 µg/mL, Merck). Cell viability of the floating cells was assayed 3 days after the co-culture using a Cell Counting Kit-8 (Dojindo, Kumamoto, Japan). The results were measured on a Multiskan™ GO microplate spectrophotometer (Thermo Fisher Scientific).

Preparation of peripheral blood and liver tissues from mice

Mouse peripheral blood samples were incubated overnight at 4 ºC, and were centrifuged at 770 × *g* for 10 min at 4 ºC to obtain serum. Mouse liver tissue samples were quickly frozen in liquid nitrogen, and were homogenized in a mixture of a T-PER^{TM} Tissue Protein Extraction Reagent, Thermo Fisher Scientific) and a proteinase inhibitor cocktail (Nacalai Tesque) with a TissueLyser LT mechanical processor (Qiagen, Venlo, Netherland). Total protein in serum and liver tissue samples was measured using a BCA protein assay (Thermo Fisher Scientific) and a Bio-Rad Protein Assay (Bio-Rad Laboratories), respectively, according to the manufacturers' instructions, and were normalized to the protein concentration.

Flow cytometric analysis

Cultured hDPSC-products (0.1 × 10^6) were suspended in a flow cytometric buffer (FCMB) at 4 °C. The FCMB consisted of Hanks' balanced salt solution (Nacalai Tesque) containing 2% heat-inactivated FBS (Equitech-Bio). The cells were incubated with R-phycoerythrin (R-PE)-conjugated primary antibodies (1 μg diluted in FCMB) at 4 °C for 45 min. As a control, R-PE-conjugated isotype-matched antibodies were used instead of the primary antibodies. The stained samples were washed with 1 mL of

ice-cold FCMB twice, and were analyzed on a FACSVerse^{TM} flow cytometer (BD Bioscience, Franklin Lake, NJ) using the FACSuite^{TM} software (BD Bioscience). The number (percentage) of positive cells was determined by comparison with the corresponding control cells stained with the corresponding isotype-matched antibody, in which a false-positive rate of less than 1% was accepted. The specific antibodies used for flow cytometric analysis are shown in **Supplementary Table 4**.

Extraction and preparation of total RNA

Cellular and tissue samples were treated with a TRIzol® reagent (Thermo Fisher Scientific). The RNA extracts were digested with a DNase I (Promega, Madison, WI), and were purified using an RNeasy® Mini Kit (Qiagen). To check for genomic DNA contamination, the RNA extracts were directly reacted with a primer pair for human glyceraldehyde-3-phosphate dehydrogenase (GAPDH; Sense, 5'-TGAAGGTCGGTGTCAACGGATTTGGC 3'; Antisense, 5'-CATGTAGGCCATGAGGTCCACCAC-3') and Quick Taq HS DyeMix (TOYOBO, Osaka, Japan) with a T-100 ^{TM} thermal cycler (Bio-Rad Laboratories). The amplification by PCR was as follows; initial activation step (95 ºC, 120 sec) and three-step cycling (95 ºC for 30 sec, 60 ºC for 30 sec, and 68 ºC for 60 sec; 30 cycles)

on a T-100^{TM} thermal cycler (Bio-Rad Laboratories). The amplified PCR products (5 μL) were separated on a 2% agarose gel (Nacalai Tesque) by gel electrophoresis on a Mupid-2plus electrophoretic machine (Mupid, Tokyo, Japan). The gels were stained with ethidium bromide (Nacalai Tesque), and were imaged on a Gel Doc^{TM} EZ System (Bio-Rad Laboratories). The total RNA was reverse-transcribed with a Revertra Ace qPCR kit (TOYOBO, Osaka, Japan), and the cDNA was obtained.

Gene expression analysis

A mixture of the cDNA (5 μg), target-specific TaqMan probes (Applied Biosystems, Foster City, CA), and TaqMan Gene Expression Master Mix (Applied Biosystems) was reacted on a real-time PCR Light Cycler 96 system (Roche, Indianapolis, IN) as follows: preincubation step 1, 50 °C for 120 sec for 1 cycle; preincubation step 2, 95 °C for 600 sec for 1 cycle; 2-step amplification, 95 °C for 120 sec and 60 °C for 60 sec for 45 cycles. Human 18S ribosomal RNA was used for normalization. The target-specific TaqMan probes are shown in **Supplementary Tables 5-7**.

Immunological and biochemical assays

The concentration of human albumin, HGF, interleukin 6, monocyte chemoattractant protein-1, sialic acid- binding Ig-like lectin-9, mouse matrix metalloproteinase 2 (MMP2), mouse MMP9, mouse tissue inhibitors of metalloproteinase 1 (TIMP1), TIMP2, and transforming growth factor beta 1 in the CM of hDPSC cultures, mouse liver tissues, and mouse peripheral serum were analyzed by ELISA using commercially available kits according to the manufacturers' protocols. Biochemical assays for alanine aminotransferase, aspartate transferase, and total bilirubin in mouse serum were analyzed using commercially available kits according to the manufacturers' protocols. Finally, the results from the immunological and biochemical tests were measured on a Multiscan^{TM} GO microplate spectrophotometer (Thermo Fisher Scientific). The commercially available kits are listed in **Supplementary Table 8**.

Preparation for histochemistry and immunohistochemistry

Mouse liver tissue samples were treated with 4% paraformaldehyde (Merck) in PBS (pH 7.4; Nacalai Tesque) for 18 hours. The samples were subsequently dehydrated in a graded ethanol bath and were cleaned in xylene. Finally, the samples were embedded in paraffin, and were cut into 6 mm-thick sections. The paraffin sections were dewaxed and were rehydrated for further histochemical and immunohistochemical studies.

Immunohistochemical analysis

Paraffin sections were incubated with 3.0 % H_2O_2 in ethanol for 30 min at room temperature to inhibit endogenous peroxidase. The sections were then treated with 10% normal mouse or rabbit serum in PBS for 60 min at room temperature. The sections were treated with primary antibodies overnight at 4 °C, followed by treatment with the EnVision+ System (Agilent Technologies, Santa Clara, CA) according to the manufacturer's instruction. Immunohistochemical negative controls were stained with non-immune isotype-matched IgG instead of the primary antibodies. Primary antibodies and isotype-matched antibodies used in immunohistochemical analysis are shown in **Supplementary Table 9**.

Supplementary Table 1. Summary of population doubling level in human deciduous pulp stem cell (hDPSC) products

Donor	P3 PDL	P10 PDL	Total PDL (final passage number)
#1	5.5	21.6	26.7 (15)
#2	5.0	21.2	33.1 (17)
#3	5.2	N/A	9.7 (7)
#4	5.9	21.7	36.5 (20)
#5	4.7	20.2	26.0 (16)
#6	4.5	21.4	32.6 (17)
#7	5.6	21.8	26.7 (14)
#8	5.2	20.2	32.3 (18)
#9	4.8	20.2	36.6 (21)
#10	5.0	20.1	34.9 (19)

P3 PDL, total population doubling level of hDPSC-products at passage 3; P10 PDL, total population doubling level of hDPSC-products at passage 10; Total PDL, total population doubling level of hDPSC-products when each donor lost the proliferative capacity

Supplementary Table 2. Summary of expression of cell surface antigens for mesenchymal stem cells in human deciduous pulp stem cell products

P3 hDPSC-products (%)

Donor	CD105	CD73	CD90	CD34	CD45	CD14	HLA-DR
#1	98.37	91.72	99.95	1.02	0.69	0.99	1.17
#2	97.17	78.27	99.50	0.96	1.09	0.98	1.67
#3	88.09	96.90	98.75	0.88	0.94	0.75	0.79
#4	89.79	41.90	89.79	0.83	0.93	0.79	0.79
#5	91.04	73.02	98.61	0.74	0.59	0.74	0.80
#6	98.76	69.66	99.39	0.94	0.90	1.17	0.96
#7	96.33	78.10	99.59	0.48	0.41	0.53	0.61
#8	89.51	91.22	99.69	1.18	1.07	0.86	1.49
#9	92.43	91.14	99.53	1.56	1.37	1.83	1.52
#10	92.57	84.48	99.51	0.77	0.83	0.48	0.80

P10 hDPSC-products (%)

Donor	CD105	CD73	CD90	CD34	CD45	CD14	HLA-DR
#1	95.09	42.35	99.57	5.92	1.13	1.40	1.34
#2	92.43	85.33	99.76	2.67	0.99	1.36	1.17
#3	N/A	N/A	N/A	N/A	N/A	N/A	N/A
#4	95.73	86.09	99.95	3.54	1.77	6.08	1.55
#5	85.73	76.09	99.95	2.54	1.37	3.08	1.35
#6	74.26	60.31	98.93	1.74	0.76	1.91	1.12
#7	63.69	94.53	99.49	2.24	1.15	1.58	1.32
#8	N/A	N/A	N/A	N/A	N/A	N/A	N/A
#9	N/A	N/A	N/A	N/A	N/A	N/A	N/A
#10	70.93	97.05	99.75	1.04	1.13	1.26	0.43

Thawed-P10 hDPSC-products (%)

Donor	CD105	CD73	CD90	CD34	CD45	CD14	HLA-DR
#1	88.23	93.82	99.27	1.19	0.81	0.94	0.93
#10	82.85	85.69	99.71	2.07	1.05	4.13	1.05

P3 hDPSC-products, passage 3 human deciduous pulp stem cell products; P10 hDPSC-products, passage 10 human deciduous pulp stem cell products; Thawed-P10 hDPSC-products, Thawed-P10 human deciduous pulp stem cell products.
HLA-DR, human leukocyte antigen DR.

Supplementary Table 3. Summary of expression of cell surface antigens for immunogenicity in human deciduous pulp stem cell products

P10 hDPSC-products (%)

Donor	HLA-ABC	HLA-G	HLA-DR	HLA-DQ	CD80	CD86	CD40	CD3
#1	89.79	1.16	1.34	1.38	4.34	3.47	2.12	1.07
#2	98.20	1.30	1.17	1.27	4.42	4.09	1.58	1.70
#3	N/A	N/A	N/A	N/A	N/A	N/A	N/A	N/A
#4	99.12	1.89	1.55	1.81	5.63	4.57	2.36	1.30
#5	99.52	1.59	1.36	1.31	3.63	3.57	1.36	1.10
#6	64.84	1.29	1.12	1.09	1.62	2.33	1.25	2.16
#7	41.48	1.30	1.32	1.08	3.46	2.12	1.36	1.61
#8	N/A	N/A	N/A	N/A	N/A	N/A	N/A	N/A
#9	N/A	N/A	N/A	N/A	N/A	N/A	N/A	N/A
#10	88.58	0.80	0.43	0.49	6.91	4.63	0.71	2.77

Thawed-P10 hDPSC-products (%)

Donor	HLA-ABC	HLA-G	HLA-DR	HLA-DQ	CD80	CD86	CD40	CD3
#2	71.89	1.00	0.93	1.15	3.46	2.12	1.36	1.61
#10	88.77	1.29	1.05	1.07	5.63	4.57	2.36	1.30

P10 hDPSC-products, passage 10 human deciduous pulp stem cell products; Thawed-P10 hDPSC-products, Thawed-P10 human deciduous pulp stem cell products.
HLA-DR, human leukocyte antigen DR.

Supplementary Table 4. Antibody information for flow cytometry.

Antibody (Clone)	Type	Manufacture
anti-CD3 antibody (HIT3a)	R-PE-conjugated, mouse IgG2b, kappa	Biolegend (San Diego, CA)
anti-CD14 antibody (63D3)	R-PE -conjugated, mouse IgG1, kappa	Biolegend (San Diego, CA)
anti-CD19 antibody (47G)	R-PE-conjugated, mouse IgG1, kappa	Biolegend (San Diego, CA)
anti-CD34 antibody (561)	R-PE-conjugated, mouse IgG2a, kappa	Biolegend (San Diego, CA)
anti-CD45 antibody (2D1)	R-PE-conjugated, mouse IgG1, kappa	Biolegend (San Diego, CA)
anti-CD56 antibody (5.1H11)	R-PE-conjugated, mouse IgG1, kappa	Biolegend (San Diego, CA)
anti-CD73 antibody (AD2)	R-PE-conjugated, mouse IgG1, kappa	Biolegend (San Diego, CA)
anti-CD80 antibody (2D10)	R-PE-conjugated, mouse IgG1, kappa	Biolegend (San Diego, CA)
anti-CD86 antibody (BU63)	R-PE-conjugated, mouse IgG1, kappa	Biolegend (San Diego, CA)
anti-CD90 antibody (5E10)	R-PE-conjugated, mouse IgG1, kappa	Biolegend (San Diego, CA)
anti-CD105 antibody (43A3)	R-PE-conjugated, mouse IgG1, kappa	Biolegend (San Diego, CA)
anti-HLA-ABC antibody (W6/32)	R-PE-conjugated, mouse IgG2a, kappa	Biolegend (San Diego, CA)
anti-HLA-DR antibody (L243)	R-PE-conjugated, mouse IgG2a, kappa	Biolegend (San Diego, CA)
anti-HLA-DQ antibody (HLADQ1)	R-PE-conjugated, mouse IgG1, kappa	Biolegend (San Diego, CA)
anti-HLA-E antibody (3D12)	R-PE-conjugated, mouse IgG1, kappa	Biolegend (San Diego, CA)
anti-HLA-G antibody (87G)	R-PE-conjugated, mouse IgG2a, kappa	Biolegend (San Diego, CA)
mouse IgG1 kappa (MOPC-21)	R-PE-conjugated	Biolegend (San Diego, CA)
mouse IgG2a kappa (MOPC-173)	R-PE-conjugated	Biolegend (San Diego, CA)
mouse IgG2b kappa (MPC-11)	R-PE-conjugated	Biolegend (San Diego, CA)

HLA, human leukocyte antigen; R-PE: R-phycoerythrin

Supplementary Table 5. TaqMan probe information for qRT-PCR assay to human adipocyte-, chondrocyte-, and osteoblast-specific genes.

Names of genes	Gene assay ID Numbers
ALP	Hs01029144_m1
BGLAP	Hs01587814_g1
COL10	Hs00166657_m1
LPL	Hs00173425_m1
PPARG	Hs0115513_m1
RUNX2	Hs00231692_m1
SOX9	Hs01001343_g1
Ribosomal RNA, 18S	Hs99999901_s1

ALP, *alkaline phosphatase* gene; *BGLAP*, *bone gamma-carboxyglutamate protein* gene; *Col10*, *collagen, type X* gene; *GAPDH*, *glyceraldehyde 3-phosphate dehydrogenase* gene; *LPL*, *lipoprotein lipase* gene; *PPARG*, *peroxisome proliferator activated receptor-gamma 2* gene; *RUNX2*, *runt-related gene 2* gene; *SOX9*, *SPY-box 9* gene.

Supplementary Table 6. TaqMan probe information for qRT-PCR assay to human hepatocyte-specific genes.

Names of genes	Gene assay ID Numbers
AFP	Hs00173490_m1
ALB	Hs00910225_m1
ARG2	Hs00982833_m1
ASL	Hs00902699_m1
ASS1	Hs01597989_g1
ATP7B	Hs00163739_m1
CPS1	Hs00157048_m1
CYP3A7	Hs00426361_m1
FAH	Hs00164611_m1
KRT18	Hs02827483_g1
NAGS	Hs00400246_m1
OTC	Hs00166892_m1
STC1	Hs00174970_m1
TF	Hs01067777_m1
TTR	Hs00174941_m1
Ribosomal RNA, 18S	Hs99999901_s1

AFP, *alpha fetoprotein* gene; *ALB*, *albumin* gene; *ARG2*, *arginase 2* gene; *ASL*, *argininosuccinate lyase* gene; *ASS1*, *argininosuccinate synthase 1* gene; ATP7B, *ATPase copper transporting beta* gene; *CPS1*, *carbamoyl-phosphate synthase 1* gene; *CYP3A7*, *cytochrome P450, family 3 subfamily A member 7* gene; *FAH*, *fumarylacetoacetate hydrolase* gene; *KRT18*, *keratin 18* gene; *NAGS*, *N-acetylglutamate synthase* gene; *OTC*, *ornithine carbamoyltransferase* gene; *STC1*, staniocalcin 1 gene; *TF*, *transferrin* gene; *TTR*, *transthyretin* gene.

Supplementary Table 7. TaqMan probe information for real-time RT-PCR

Human

Gene	ID Number
STC1	Hs00174970_g1
Ribosomal RNA, 18S	Hs99999901_s1

STC1, stanniocalcin 1.

Mouse

Gene	ID Number
Acta2	Mm00725412_s1
Col1a1	Mm00801666_g1
Mmp2	Mm00439498_m1
Mmp9	Mm00442991_m1.
Timp1	Mm01341361_m1
Timp2	Mm00441825_m1
Tgfb1	Mm01178820_m1
18S	Mm03928990_g1

Acta2, *actin, alpha 2, smooth muscle, aorta*; *Col1a1*, *collagen type I alpha 1 chain*; *Mmp2*, *matrix metalloprotein 2*; *Mmp9*, *matrix metalloprotein 9*; *TIMP1*, *TIMP metallopropeptidase inhibitor 1*; *TIMP2*, *TIMP metallopropeptidase inhibitor 2*.

Supplementary Table 8. Commercially available kit information for biochemical analysis and enzyme-labeled immunosorbent assay.

Target	Product	Manufacture
ALT	Transaminase CII-Test	Wako Pure Chemicals (Tokyo, Japan)
AST	Transaminase CII-Test	Wako Pure Chemicals (Tokyo, Japan)
Bilirubin, total	Bilirubin QuantiChrom Assay Kit	BioAssay Systems (Hayward, USA)
Albumin, human	Human Albumin EIA Kit	TaKaRa Bio (Shiga, Japan)
HGF, human	Human HGF Quantikine ELISA Kit	R&D Systems (Minneapolis, MN)
IL6, human	Human IL-6 Quantikine ELISA Kit	R&D Systems (Minneapolis, MN)
MCP1, human	Human CCL2/MCP-1 Quantikine ELISA Kit	R&D Systems (Minneapolis, MN)
MMP2, mouse	Mouse Matrix Metalloproteinase 2 ELISA Kit	BlueGene Biotech (Shanghai, China)
MMP9, mouse	MMP9 ELISA Kit	Dldevelop (Jiangsu, China)
SIGLEC9	SIGLEC9 Human ELISA Kit	Thermo Fisher Scientific (Waltham, MA)
TGFβ1, mouse	ELISA Kit for Transforming Growth Factor Beta 1	Cloud-Clone (Houston, TX, USA)
TIMP1, mouse	TIMP1 ELISA Kit	Dldevelop (Jiangsu, China)
TIMP2, mouse	TIMP ELISA Kit	Dldevelop (Jiangsu, China)

ALT, alanine aminotransferase; AST: aspartic aminotransferase; HGF, hepatocyte growth factor; IL6, interleukin 6; MCP1, monocyte chemotactic protein 1; MMP2, matrix metalloproteinase 2; MMP9, matrix metalloproteinase 9; SIGLEC9, sialic acid-binding Ig-like lectin 9; TGFβ1, Transforming growth factor beta; TIMP1, tissue inhibitor of matrix proteinases 1; TIMP1, tissue inhibitor of matrix proteinases 2.

Supplementary Table 9. Antibody information for immunohistochemistry.

Antibody (Clone)	Type	Manufacture
anti-ACTA2 antibody	purified, rabbit IgG	Abcam (Cambridge, England)
anti-albumin, human, antibody (15C7)	purified, mouse IgG2b, kappa	Abcam (Cambridge, England)
anti-HepPar1 antibody (OCH1E5)	purified, mouse IgG1, kappa	Agilent Technologies (Santa Calara, CA)
Anti-HLA-ABC antibody (W6/32)	purified, mouse IgG2a, kappa	Agilent Technologies (Santa Calara, CA)
mouse IgG1 kappa (MOPC-21)	purified,	Biolegend (San Diego, CA)
mouse IgG2a kappa (MOPC-173)	purified,	Biolegend (San Diego, CA)
mouse IgG2b kappa (MPC-11)	purified,	Biolegend (San Diego, CA)
rabbit IgG	purified	Biolegend (San Diego, CA)

ACTA2, actin, alpha 2, smooth muscle, aorta; HepPar1, hepatocyte paraffin 1; HLA-ABC, human leukocyte antigens A, B, and C.

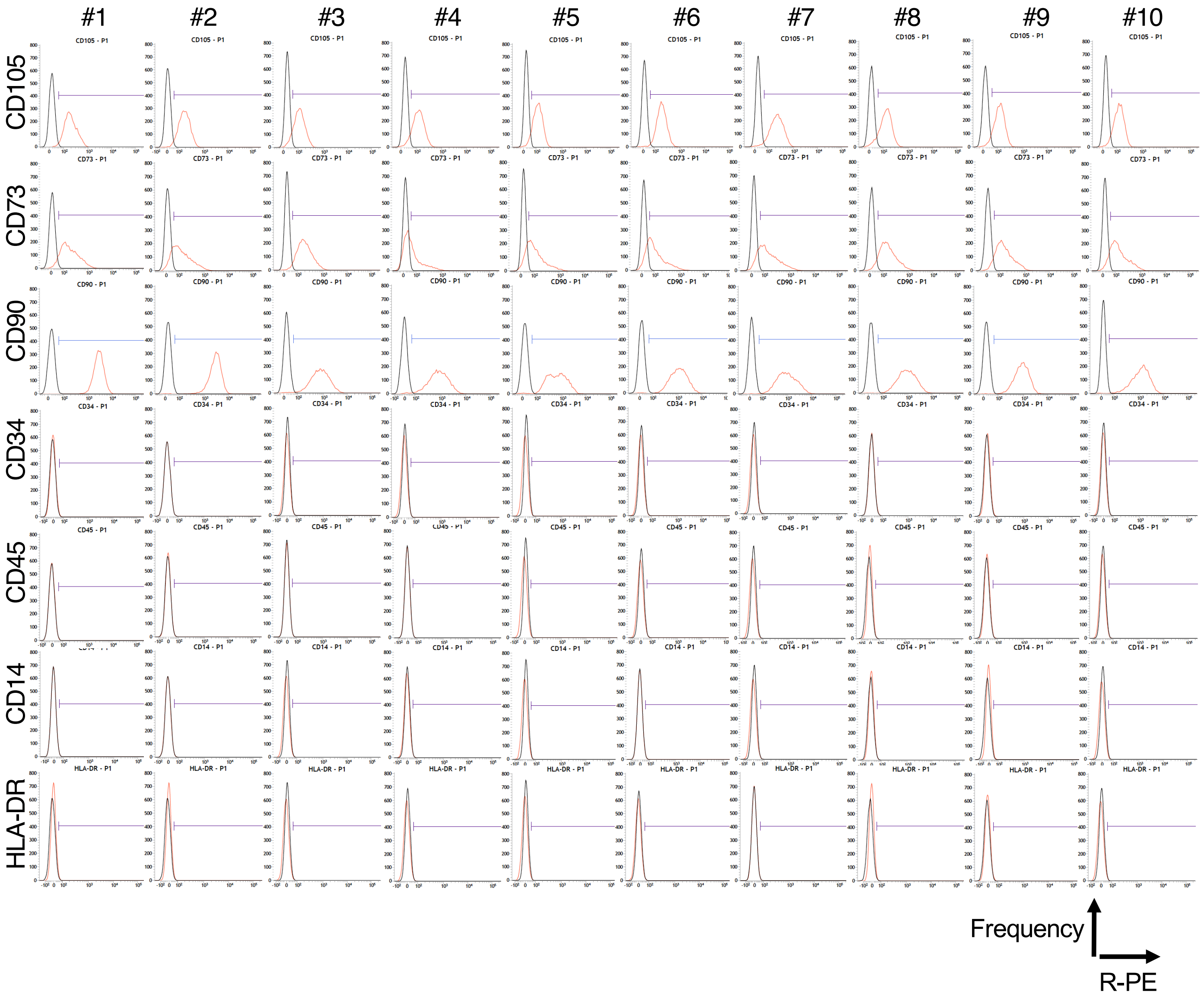

Supplementary Figure 1. Cell surface marker analysis of the P3 hDPSCs was tested by flow cytometric assay. The results are shown as representative histograms. The red- and black-colored histograms indicate the frequency stained with target antigen-specific and isotype-matched antibodies, respectively. R-PE, R-phycoerythrin.

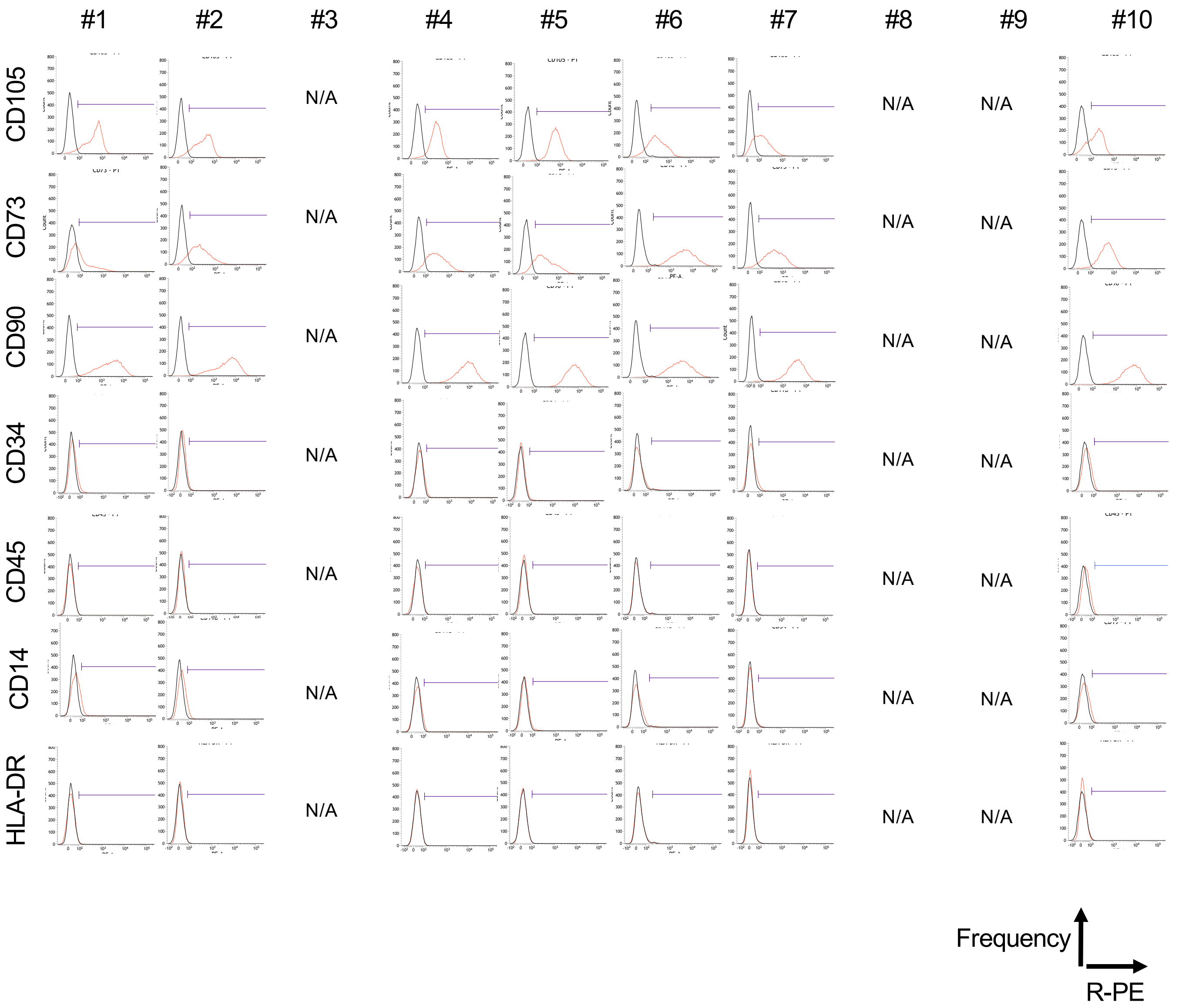

Supplementary Figure 2. Cell surface marker analysis of the P10 hDPSCs was tested by flow cytometric assay. The results are shown as representative histograms. The red- and black-colored histograms indicate the frequency stained with target antigen-specific and isotype-matched antibodies, respectively. R-PE, R-phycoerythrin.

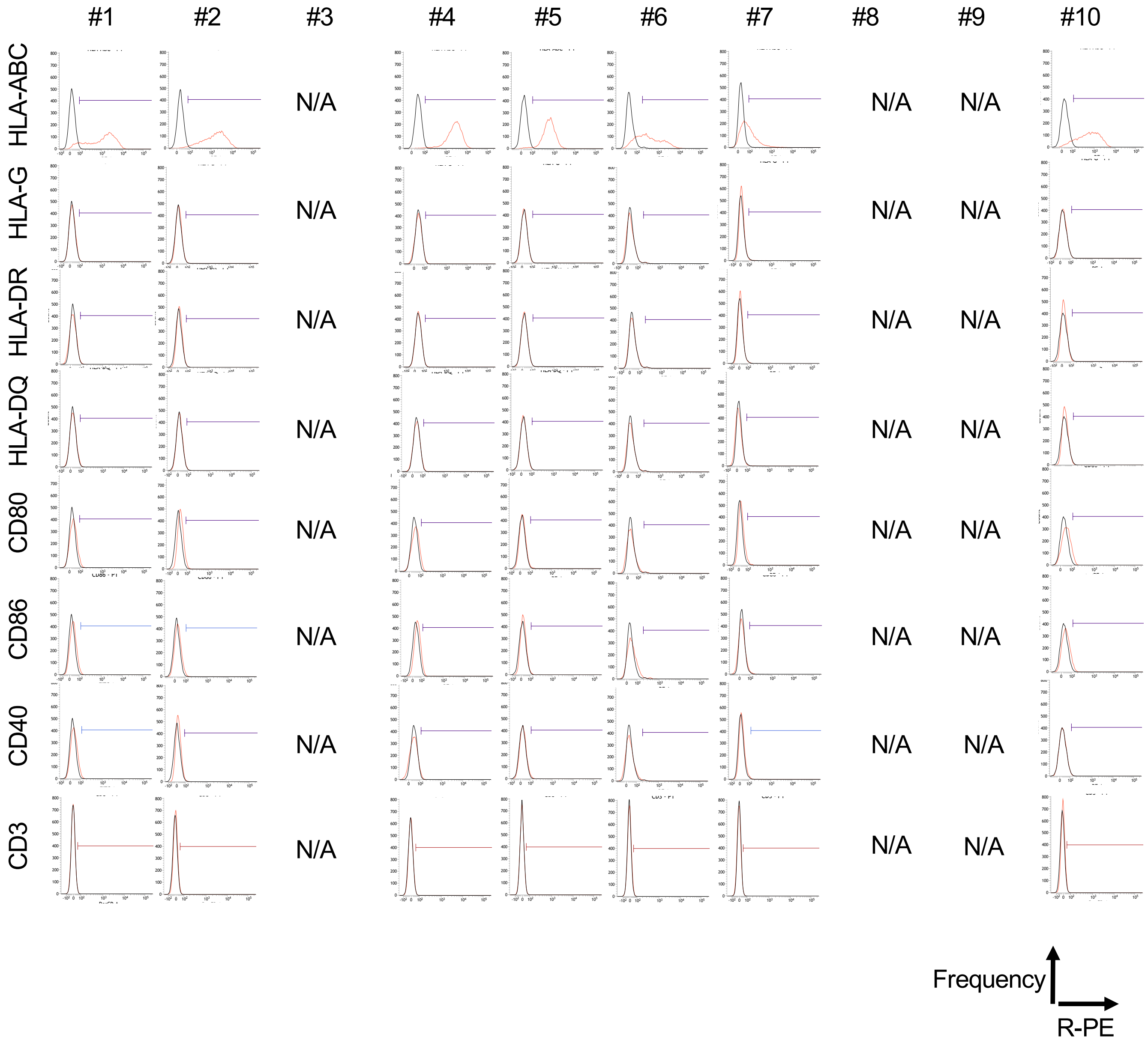

Supplementary Figure 3. Cell surface marker analysis of the P10 hDPSCs was tested by flow cytometric assay. The results are shown as representative histograms. The red- and black-colored histograms indicate the frequency stained with target antigen-specific and isotype-matched antibodies, respectively. R-PE, R-phycoerythrin.

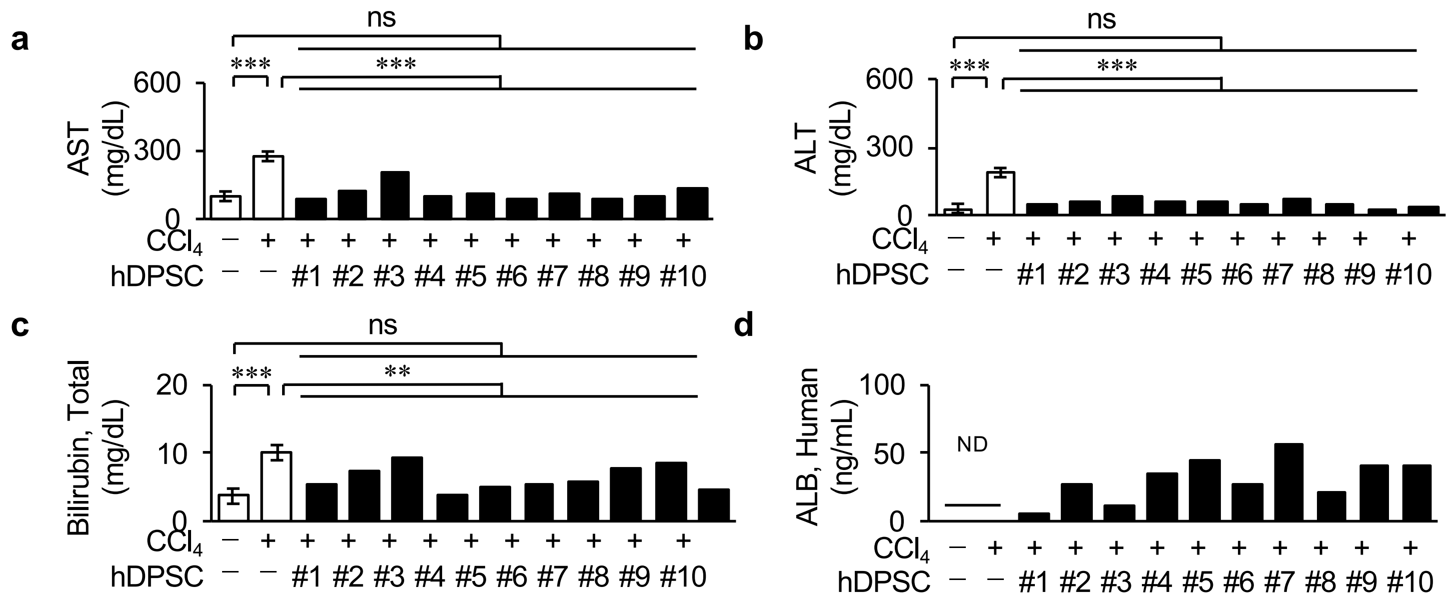

Supplementary Figure 4. Transplantation of P3 hDPSC-products improved chronically CCl_4-damaged liver dysfunction in mice. Each donor-derived P3 hDPSC-products were intrasplenically transplanted into 4-week-CCl_4-treated immunocompetent mice without immunosuppressant (n = 5). The age-matched control non-CCl_4-treated (n = 5) and non-transplanted CCl_4-treated (n = 5) immunocompetent mice were used as experimental controls. (**a**(**d**), and human albumin (ALB, **h**) were examined by biochemical assays and ELISA. **a-d**: n = 5 for all groups. $**P < 0.01$, $***P < 0.005$. The graph bars show the mean ± SEM (white columns) or the mean (black columns). ND, not detected; ns, not significant.

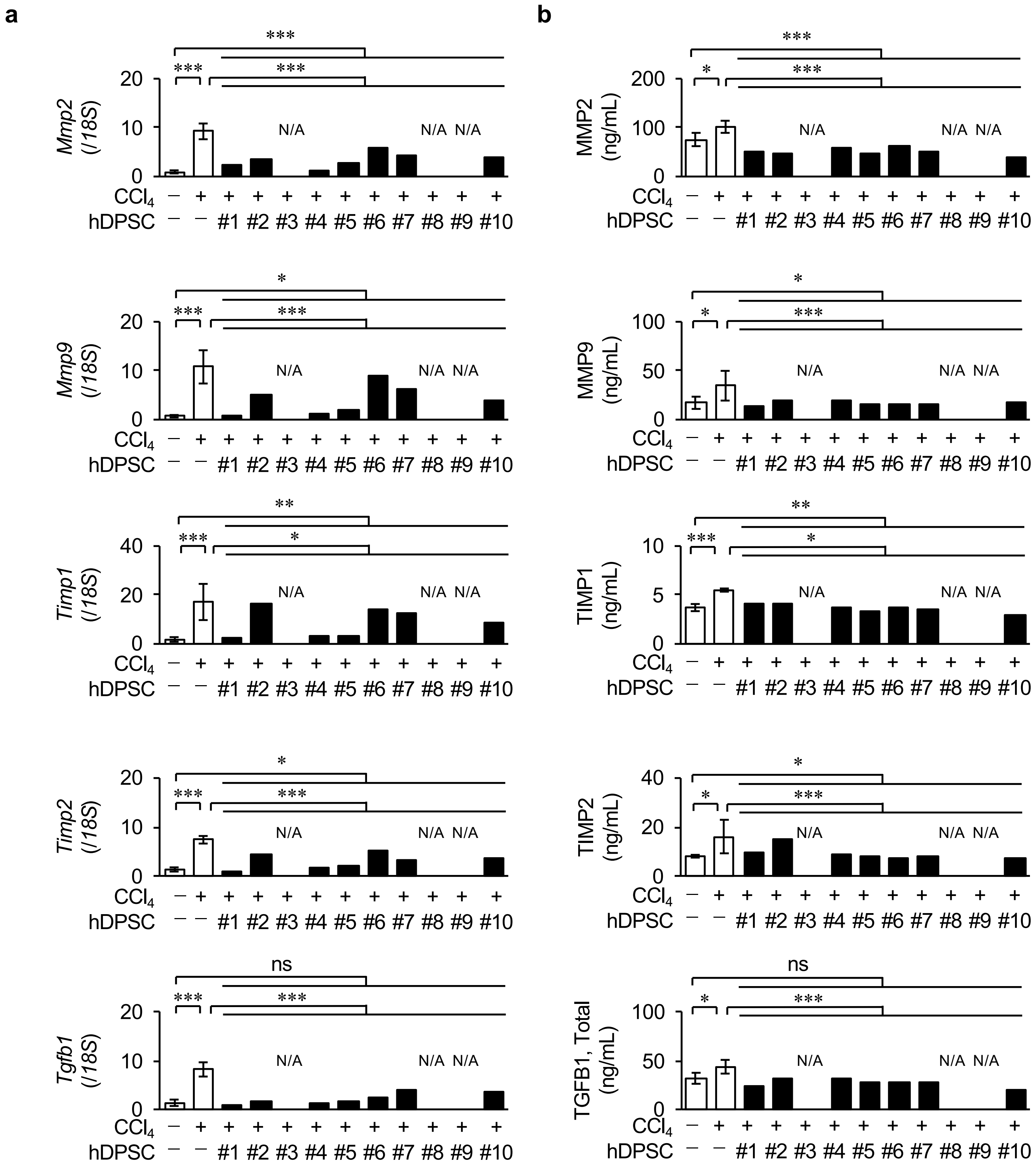

Supplementary Figure 5. Anti-fibrotic effects of P10 MCB-hDPSC transplantation in mouse CCl_4-induced fibrotic liver. Figure legend of this figure was described on next page.

Supplementary Figure 5. Anti-fibrotic effects of P10 hDPSC-transplantation in mouse CCl_4-induced fibrotic liver. P10 hDPSC-products except for donor #3, #8, and #9 were intrasplenically transplanted into 4-week-CCl_4-treated immunocompitent mice without immunosuppressant. (**a**) Expression of mouse *matrix metalloprotease 2* (*Mmp2*), *Mmp9*, *tissue inhibitor of metallopropeptidase 1* (*Timp1*), *Timp2*, and *transforming growth factor beta 1* (*Tgfb1*) genes in mouse liver tissues was analyzed by quantitative reverse transcription polymerase chain reaction assay. The results were shown as the ratios to the expression of 18S ribosomal RNA (*18S*). (**b**) Expression of mouse MMP2, MMP9, TIMP1, TIMP2, and TGFB1 proteins in mouse liver tissues was analyzed by enzyme-linked immuno-sorbent assay. n = 5 for all groups. $*P < 0.05$, $***P < 0.005$. The graph bars show means ± SEM (white columns) or means (black columns). N/A, not available; ns, not significant.

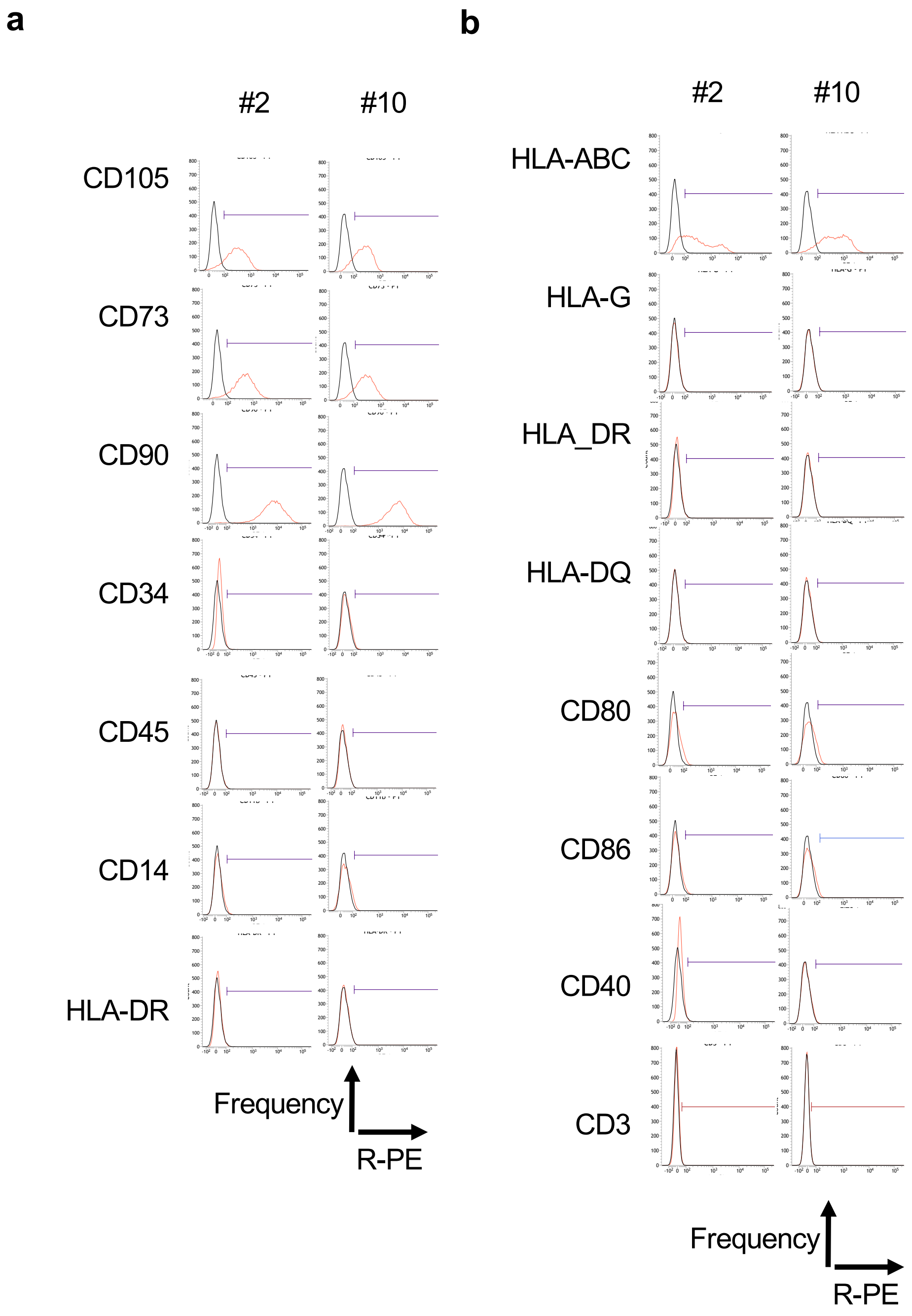

Supplementary Figure 6. Cell surface marker analysis of the P10 hDPSCs was tested by flow cytometric assay. Markers for mesenchymal stem cells (**a**) and immunogenic antigen (**b**) were assessed in the final hDPSC-products. The results are shown as representative histograms. The red- and black-colored histograms indicate the frequency stained with target antigen-specific and isotype-matched antibodies, respectively. R-PE, R-phycoerythrin.

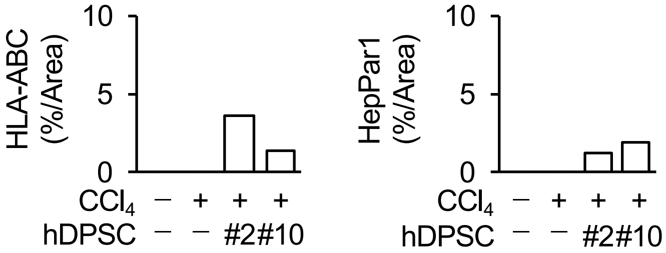

Supplementary Figure 7. Transplantation of final hDPSC-products from WCB improves CCl_4-induced pro-fibrotic markers in mice. Distribution of the donor cells and deposition of fibrous tissues in the CCl_4-damaged fibrotic liver tissues was investigated by immunohistochemical assay and collagen staining. The results are shown as the ratios of the HLA-ABC- and HepPar1- positive areas in the mouse liver tissue.
